# Supplementary material for: CDK4/6 inhibition enhances CAR-T cell therapy in solid tumors
Source: Mol Ther. 2026 Mar 24;34(7):4021–34. doi: 10.1016/j.ymthe.2026.03.024 (PMC13330056; doi:10.1016/j.ymthe.2026.03.024)
Supplement: Supplementary file 1 [file mmc1.pdf]

YMTHE, Volume *34*

Supplemental Information

CDK4/6 inhibition enhances

CAR-T cell therapy in solid tumors

Emily J. Lelliott, Jonathan Naddaf, Kun-Hui Lu, Katherine D. Cummins, Kelly M. Ramsbottom, David S. Reynolds, Michael Taylor, Krutika Ambani, Isabelle Munoz, Susan Jackson, Jessica Li, Cheok Weng Chan, Kara L. Britt, Paul A. Beavis, Shom Goel, and Jane Oliaro

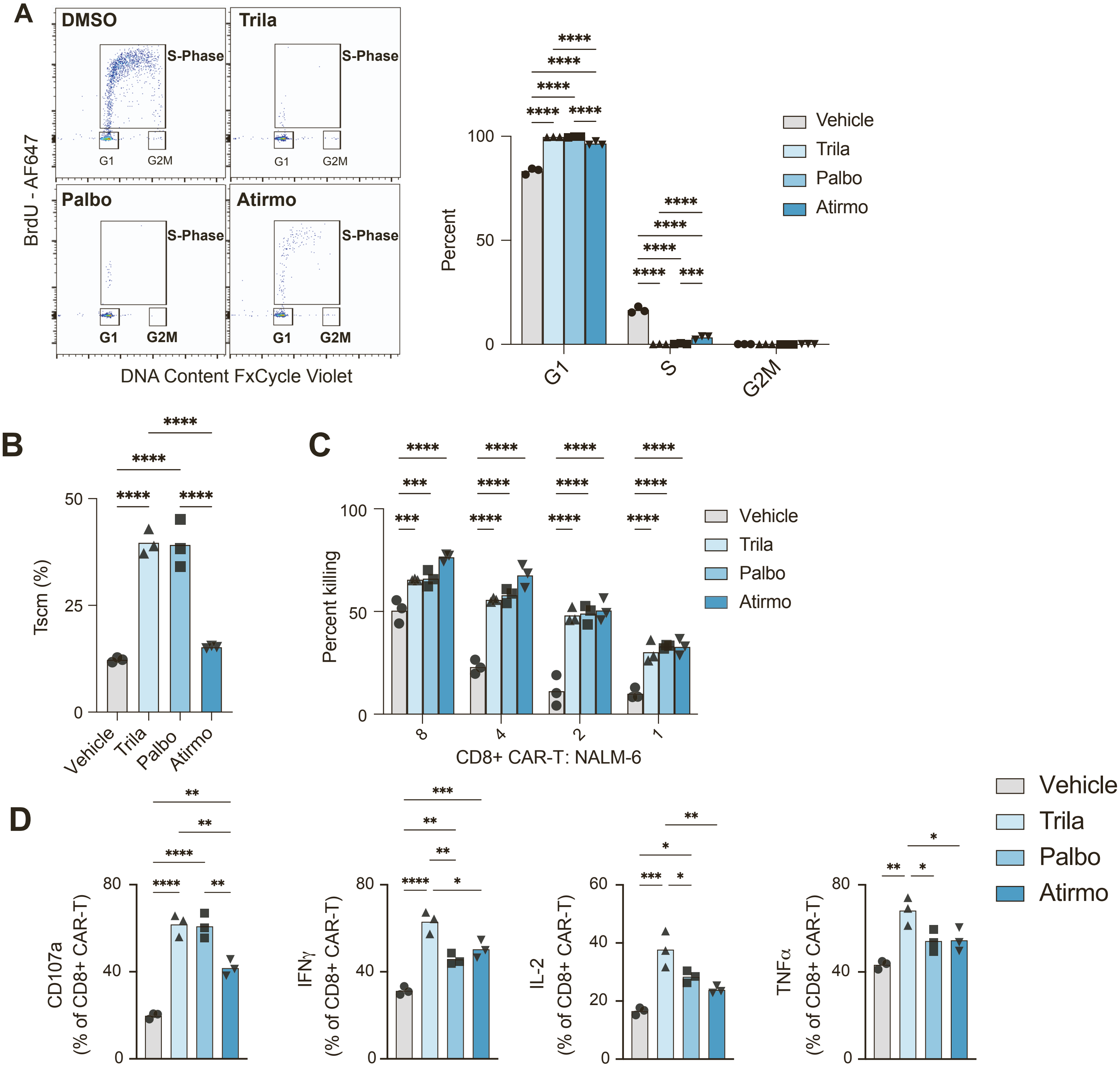

Figure S1. Human T cells were isolated from healthy donors, activated, and transduced to express a CD19-directed CAR. Transduced cells were then cultured for 6 days in trilaciclib, palbociclib, atirmociclib or DMSO (vehicle) as described in Figure 1A. (A) Cell cycle analysis using BrdU incorporation. BrdU was added to drug treated cells in culture 1 hour prior to harvest and analysis by flow cytometry. Comparisons were made using Ordinary two-way ANOVA Tukey's multiple comparisons test, n=3. (B) Drug-treated CAR-T cells were harvested and the frequency of stem cell memory T cells (Tscm) was determined by flow cytometry using gating shown in Figure 1A. Comparisons were made using Ordinary one-way ANOVA Tukey's multiple comparisons test, n=3. (C) CAR-T cell killing activity against NALM6 tumor cell targets. CAR-T cells were co-cultured with 51Cr-labelled NALM6 tumor cells at increasing ratios of CD8+ CAR-T cells to NALM6 tumor cells. Specific lysis was measured by percent chromium release and relative killing was calculated by normalising vehicle control to maximum killing of 100%. Comparisons made using Ordinary two-way ANOVA Tukey's multiple comparisons test, n=3. (D) Degranulation (CD107a) and cytokine production by CAR-T cells co-cultured with NALM6 tumor cells in vitro for 4 h. Fluor-conjugated CD107a-targeting antibody and Golgi plug was added to co-cultures before staining for analysis by flow cytometry. Comparisons made using Ordinary two-way ANOVA Tukey's multiple comparisons test, n=3. Error bars show +/- SEM. *p<0.05, **p<0.01, ***p<0.001, ****p<0.0001.

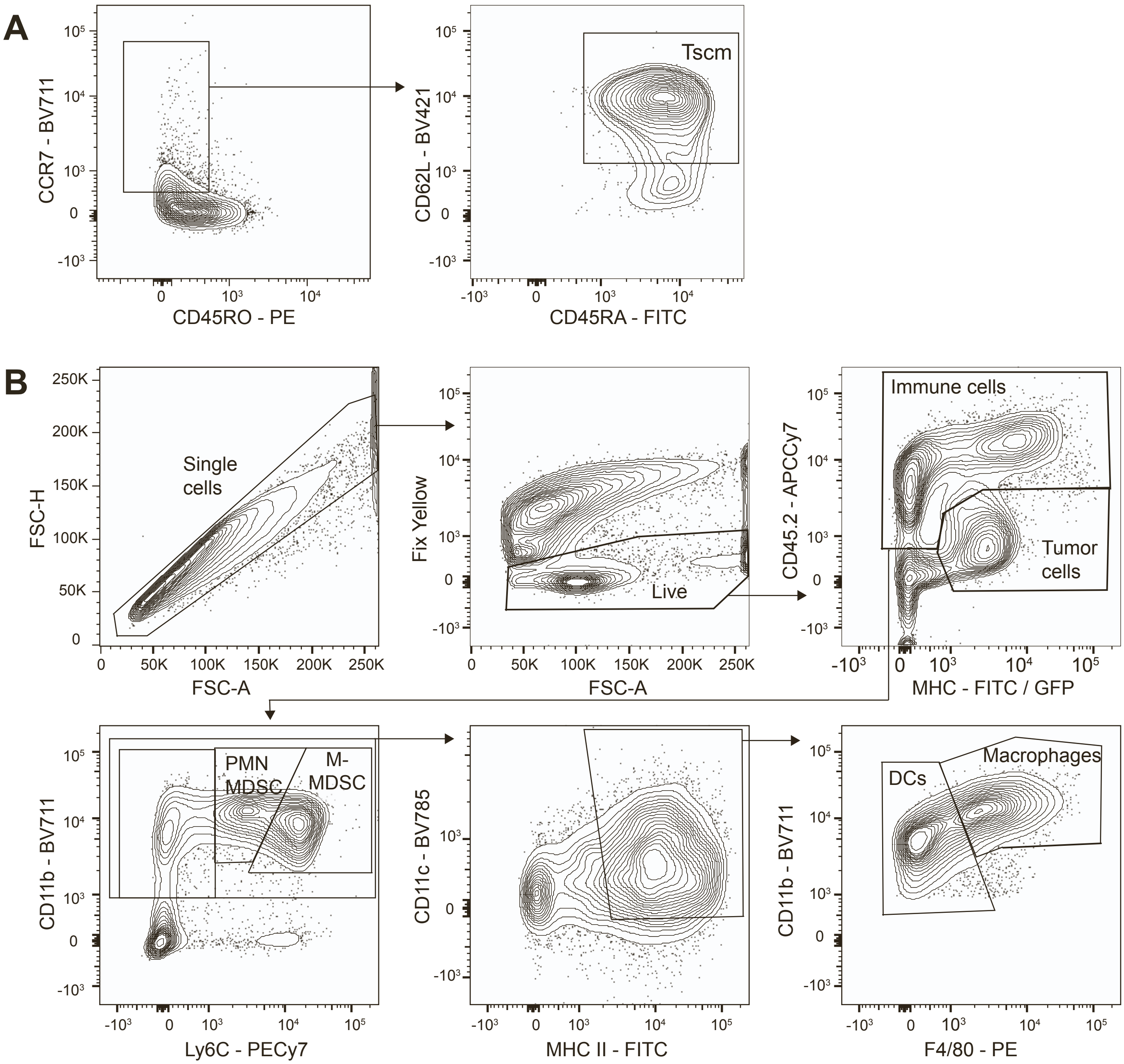

Figure S2. Flow cytometry gating. (A) Gating for human stem cell memory T cells (Tscm) first gated on CD8+ T cells. (B) Gating for tumor cells myeloid subsets from GFP+ E0771-hHER2 tumors.

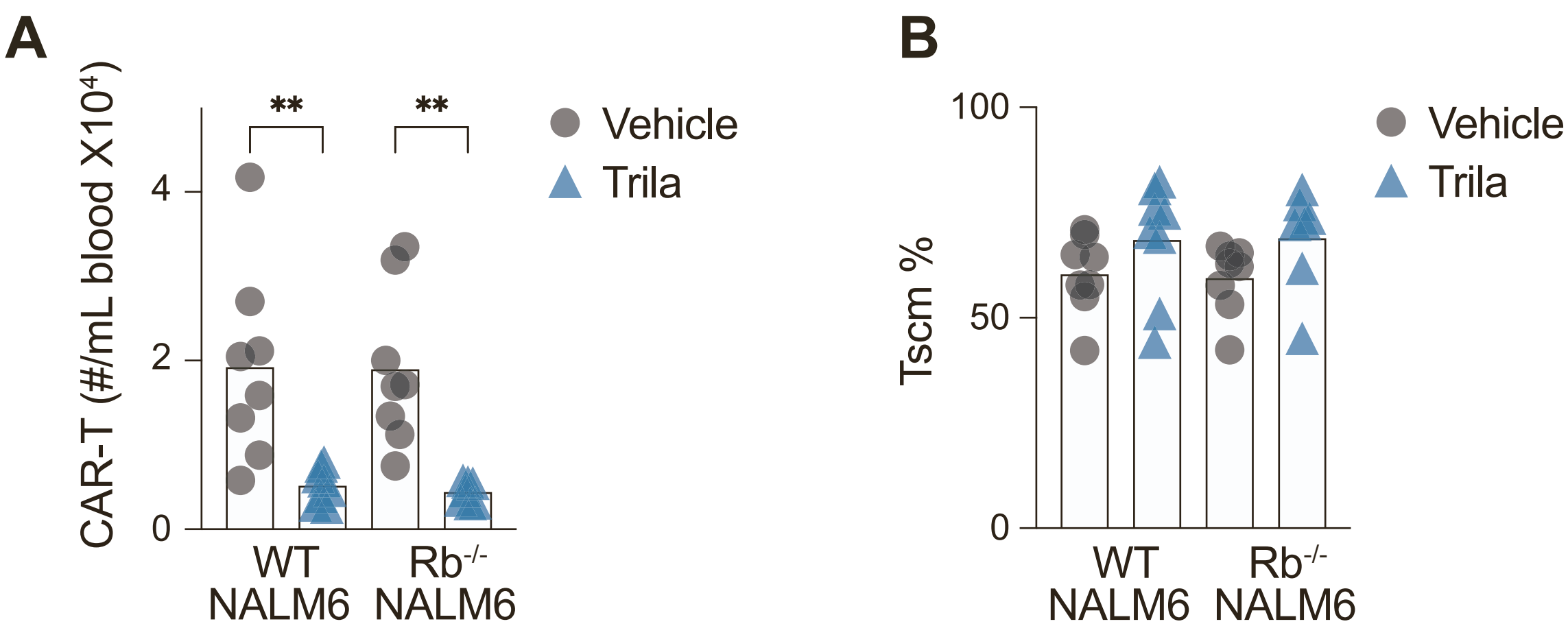

Figure S3. CD19-directed human CAR-T cells were transferred into NSG mice bearing wildtype (WT) or Rb deficient (Rb-/-) NALM6 blood cancer. Mice were treated with continuous 3 days on/4 days off trilaciclib regimen starting the day of CAR-T cell transfer. Blood was collected after two cycles of treatment. (A) CAR-T cell numbers and (B) frequency of CD8+ stem cell memory CAR-T cells (Tscm) were determined by flow cytometry. Comparisons made using Ordinary one-way ANOVA Tukey's multiple comparisons test, n=3. Error bars show +/- SEM. **p<0.01.

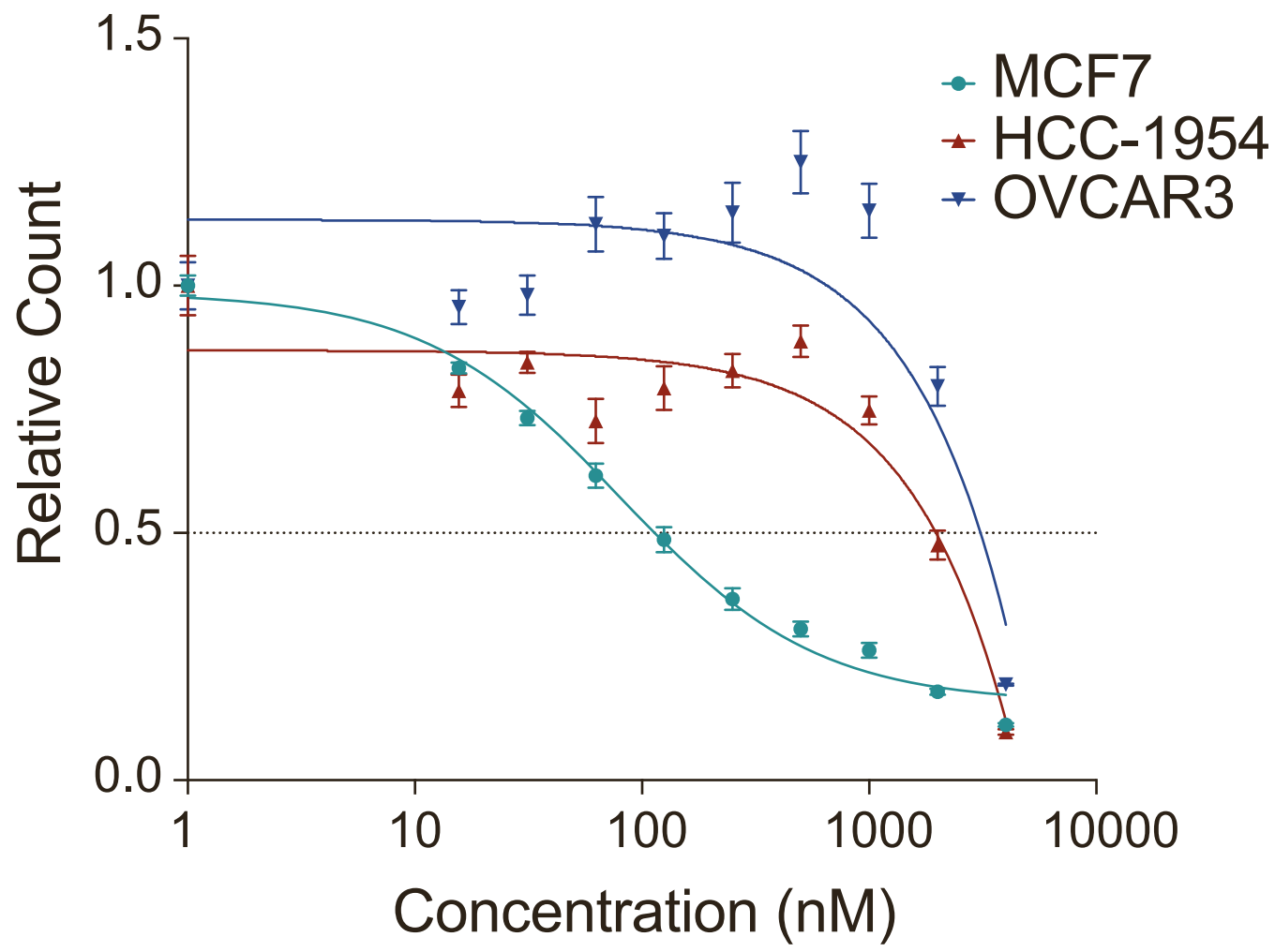

Figure S4. MCF7, OVCAR3, and HCC1954 cells were treated with a ten-point dose concentration from 15.625 nmol/L to 4000 nmol/L trilaciclib for 72 hours to determine dose response.

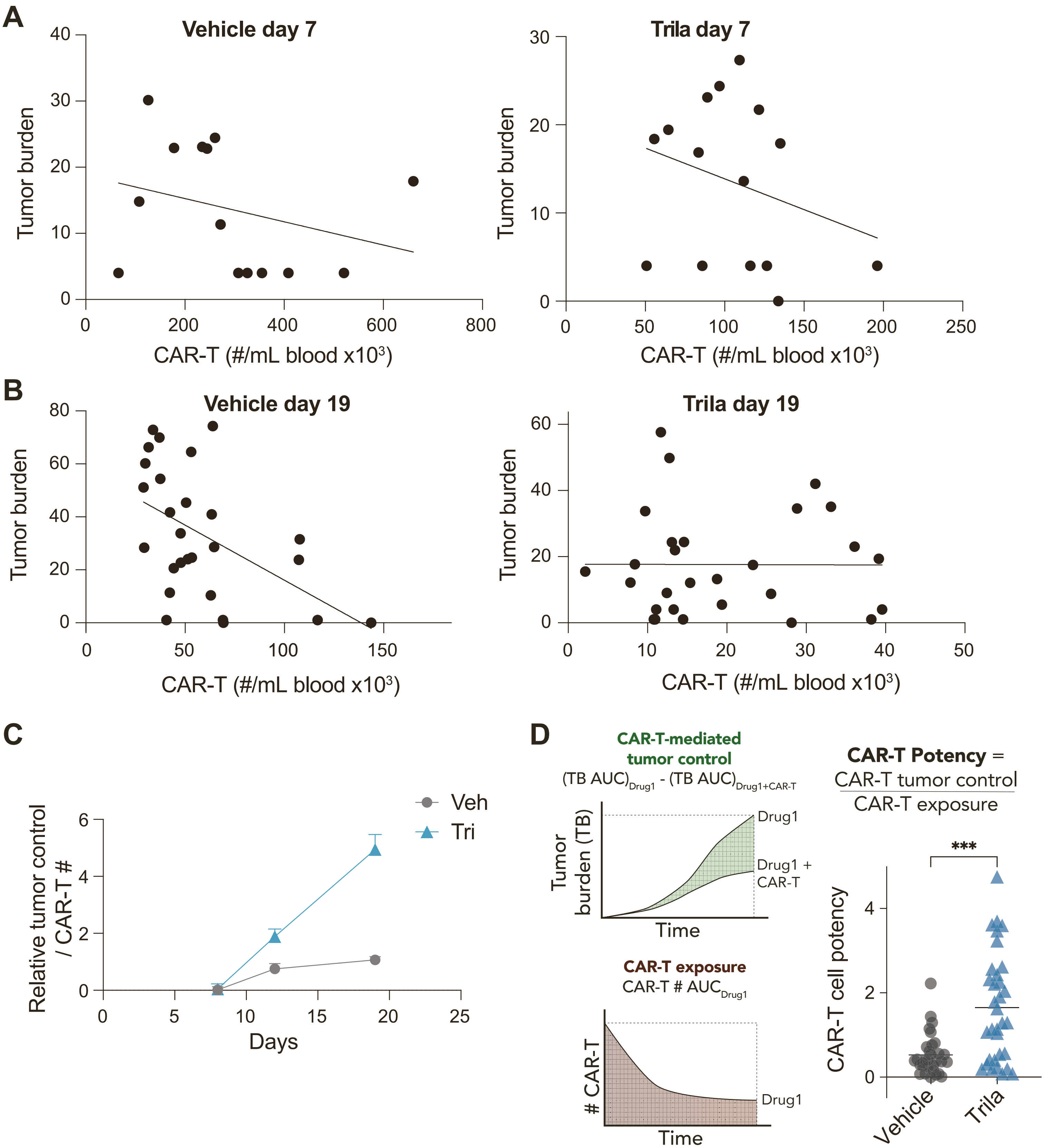

Figure S5. (A-B) Correlation between circulating CAR-T cell numbers and tumor burden at indicated time points in vehicle- and trilaciclib-treated E0771-hHER tumor bearing mice receiving hHER2-directed CAR-T cells. (C) Per-cell CAR-T anti-tumor potency over time, calculated by normalizing tumor burden in each combination-treated mice to the average tumor burden of matched drug-only controls, and dividing by CAR-T cell numbers in the blood for the same mouse at the corresponding time point. (D) Integrated measure of overall CAR-T cell potency, calculated as cumulative CAR-T–mediated tumor control (difference in tumor AUC relative to drug-only controls) divided by cumulative CAR-T cell exposure (CAR-T cell AUC). Comparisons made using Mann-Whitney nonparametric test, n=31-33, ***$p<0.001$.

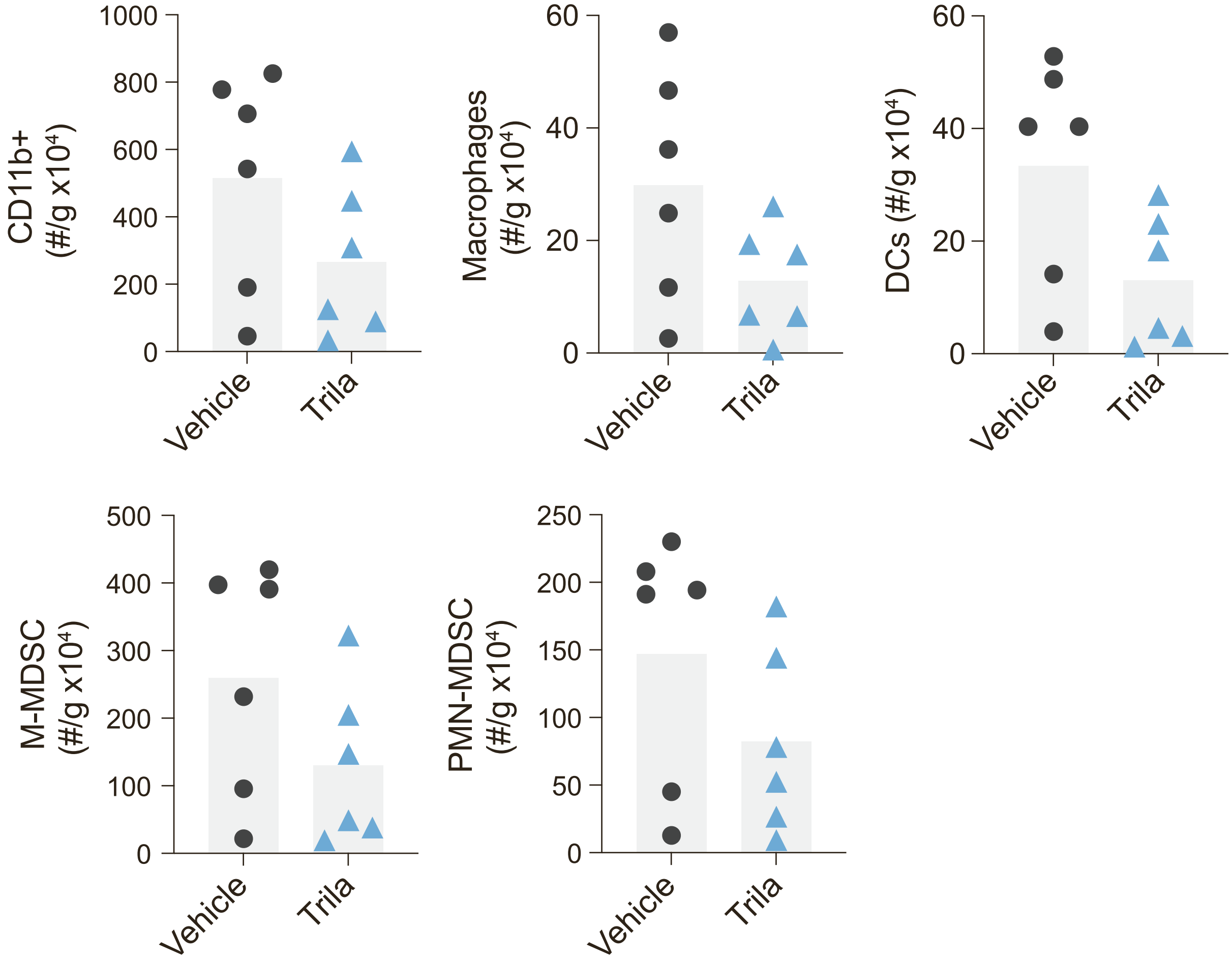

Figure S6. HER2-directed mouse CAR-T cells were transferred into immunocompetent C57BL/5 mice 5 days post mammary fat pad inoculation with the mouse breast cancer cell line E0771-hHER2. Mice were treated with continuous 3 days on/4 days off trilaciclib regimen starting the day of CAR-T cell transfer. Tumors were harvested following two cycles of treatment. Total number of myeloid populations was determined by flow cytometry using gating shown in Figure S1B. No statistically significant differences observed by Mann-Whitney nonparametric t-test.
