# Supplementary material for: CDK4/6 inhibition enhances CAR-T cell therapy in solid tumors
Source: Mol Ther. 2026 Mar 24;34(7):4021–34. doi: 10.1016/j.ymthe.2026.03.024 (PMC13330056; doi:10.1016/j.ymthe.2026.03.024)
Supplement: Supplementary file 2 [file mmc2.pdf]

Molecular Therapy
Original Article

CDK4/6 inhibition enhances CAR-T cell therapy in solid tumors

Emily J. Lelliott,[1,4] Jonathan Naddaf,[1] Kun-Hui Lu,[2,4] Katherine D. Cummins,[1] Kelly M. Ramsbottom,[1] David S. Reynolds,[1,4] Michael Taylor,[1] Krutika Ambani,[2] Isabelle Munoz,[1,4] Susan Jackson,[3] Jessica Li,[1] Cheok Weng Chan,[1] Kara L. Britt,[3,4] Paul A. Beavis,[1,4] Shom Goel,[2,4,5] and Jane Oliaro[1,4,5]

[1]Cancer Immunology Program, Peter MacCallum Cancer Centre, Melbourne, VIC 3000, Australia; [2]Cancer Biology and Therapeutics Program, Peter MacCallum Cancer Centre, Melbourne, VIC 3000, Australia; [3]Cancer Evolution and Metastasis Program, Peter MacCallum Cancer Centre, Melbourne, VIC 3000, Australia; [4]Sir Peter MacCallum Department of Oncology, The University of Melbourne, Parkville, VIC 3010, Australia

CDK4/6 inhibitors promote anti-tumor immunity through diverse mechanisms, positioning them as promising adjuvants to cancer immunotherapies. While CDK4/6 inhibitors have demonstrated strong synergy with immune checkpoint inhibitors across numerous preclinical cancer models, their combination with CAR-T cell therapy remains unexplored. In this study, we examined the efficacy of combined CDK4/6 inhibition (trilaciclib) and CAR-T therapy across a range of preclinical blood and solid cancer models. *In vitro*, trilaciclib enhanced human CAR-T cell cytotoxicity and metabolic fitness while reducing expansion. *In vivo*, the combination outperformed single agents against retinoblastoma protein (RB)-proficient, trilaciclib-sensitive CD19+ leukemia. However, in an equivalent RB-deficient model, the combination therapy was no more effective than CAR-T cells alone, suggesting that enhanced CAR-T cell function may be offset by reduced expansion. In contrast, in solid cancer models the combination was consistently more efficacious than either monotherapy. Notably, combination effects were most pronounced in immunocompetent mouse models, including a model with poor sensitivity to trilaciclib as a monotherapy. Mechanistically, CDK4/6 inhibition reduced tumor-infiltrating T-regulatory cells while enhancing CD8+ CAR-T cell persistence, tumor trafficking, and cytotoxic function within the tumor. Together, these findings suggest that trilaciclib and CAR-T cell therapy may be an effective combinatorial treatment for solid cancers.

INTRODUCTION

The advent of immune checkpoint inhibitors (ICIs) marked a breakthrough in cancer care, becoming the first cancer immunotherapy to be widely adopted as a treatment for a variety of malignancies. More recently, chimeric antigen receptor (CAR)-T cell therapy has emerged as a new class of cancer immunotherapy. CAR-T cell therapy has achieved major clinical success in the treatment of a subset of aggressive blood cancers but exhibits notoriously poor efficacy against solid tumors.[1,2] This is predominately due to the additional challenges imposed by solid tumors, such as an inability of CAR-T cells to traffic efficiently to tumors, coupled with the hostile and immunosuppressive solid tumor microenvironment.[3] Although both ICI and CAR-T cell therapies are revolutionary new pillars in cancer treatment, a large proportion of patients fail to respond to these treatments, or relapse following an initially successful response.[4–6] These low initial response rates combined with high relapse rates have underscored an urgent need to explore novel strategies to improve the efficacy and durability of existing cancer immunotherapies.

Small molecule inhibitors of cyclin dependent kinases 4 and 6 (CDK4/6) are a modern class of cytostatic cancer therapy, which function by blocking proliferation of cycling tumor cells via activation of the CDK4/6-regulated tumor-suppressive retinoblastoma protein (RB).[7] In addition to direct blockade of tumor cell proliferation, CDK4/6 inhibitors also elicit their therapeutic activity through the induction of an anti-tumor immune response.[8] This occurs through a diverse spectrum of mechanisms, such as enhancing the immunogenicity of tumors cells,[8–10] augmenting cytotoxic T cell memory and function,[11–13] and depleting tumor-infiltrating regulatory T cells.[8,11,14] Collectively, the immunomodulatory effects of CDK4/6 inhibitors position them as promising adjuvants for enhancing cancer immunotherapy. Indeed, we and others have shown these inhibitors synergize with ICI in various preclinical tumor models,[8–11] which has led to ongoing exploration of this combination across multiple clinical trials. However, while the combination of CDK4/6 inhibition and ICI has been extensively studied, the potential of pairing CDK4/6 inhibition with CAR-T cell therapy has not yet been explored. Given the multifaceted effects of CDK4/6

Received 19 July 2025; accepted 16 March 2026;
https://doi.org/10.1016/j.ymthe.2026.03.024.

[5]These authors contributed equally

Correspondence: Dr Emily Lelliott, Cancer Immunology Program, Peter MacCallum Cancer Centre, Melbourne, VIC 3000, Australia.
E-mail: emily.lelliott@petermac.org
Correspondence: Prof Shom Goel, Cancer Biology and Therapeutics Program, Peter MacCallum Cancer Centre, Melbourne, VIC 3000, Australia.
E-mail: shom.goel@petermac.org
Correspondence: Prof Jane Oliaro, Cancer Immunology Program, Peter MacCallum Cancer Centre, Melbourne, VIC 3000, Australia.
E-mail: jane.oliaro@petermac.org

Published by Elsevier Inc. on behalf of The American Society of Gene and Cell Therapy.
This is an open access article under the CC BY license (http://creativecommons.org/licenses/by/4.0/).

inhibitors on endogenous immunity, we hypothesized that pharmacological inhibition with CDK4/6 would enhance the therapeutic benefit of CAR-T cell therapy. The aim of this study was therefore to examine the efficacy and mechanistic basis of combined CAR-T therapy and the CDK4/6 inhibitor, trilaciclib, across several preclinical models of blood and solid malignancies.

RESULTS

CDK4/6 inhibition enhances anti-tumor cytotoxicity and metabolic fitness of human CAR-T cells *in vitro*

CD19-targeted CAR-T cells are the most widely used approved cellular therapy, demonstrating exceptional clinical efficacy against various CD19-positive blood cancers. Therefore, to begin investigating the potential combinatorial efficacy of CDK4/6 inhibition and CAR-T cell therapy, we first utilized a model of CD19-positive human B cell acute lymphoblastic leukemia, NALM-6. To examine the direct effects of CDK4/6 inhibition on human CAR-T cells, T cells were isolated from healthy donors and virally transduced to express a CD19-directed CAR, in line with clinical manufacturing protocols. The CD19 CAR-T cells were then cultured for 7 days in the presence of the CDK4/6 inhibitor, trilaciclib, or a vehicle control, after which their cytotoxic activity, metabolic fitness, and expansion were measured (Figure 1A).

To examine cytotoxic potential, vehicle- or trilaciclib-treated CD19 CAR-T cells were washed and co-cultured with NALM-6 tumor cells for 4 h. CAR-T cells pre-treated with trilaciclib showed a marked increase in cytotoxic capacity compared with vehicle controls (Figures 1B–1D). Specifically, CAR-T cells cultured in trilaciclib demonstrated on average a 6.4-fold increase in killing efficiency against NALM-6 target cells compared with those cultures in vehicle (Figure 1B), coupled with significantly higher levels of degranulation (Figure 1C) and interferon-gamma (IFNγ) production (Figure 1D) upon target recognition.

We have previously reported that CDK4/6 inhibitors act directly on T cells to promote their differentiation into memory-like cells with superior *in vivo* persistence.[11,12] A key functional feature of memory T cells is enhanced mitochondrial metabolism and spare respiratory capacity.[15,16] This represents the capacity for a cells' mitochondria to generate additional energy when required,[17] such as that needed to support the longevity and recall capacity of memory T cells. To determine the effects of trilaciclib on the spare respiratory capacity of CAR-T cells, vehicle- or trilaciclib-treated CD19 CAR-T cells were washed and subjected to a mitochondrial stress test, as previously described.[18] Consistent with an induction in T cell memory, trilaciclib-treated CD19 CAR-T cells exhibited a significant increase in basal mitochondrial metabolism (oxidative phosphorylation) and spare respiratory capacity as measured by cellular oxygen consumption rate (Figures 1E–1G).

In addition to cytotoxic and metabolic potential, CAR-T cell expansion in response to target recognition is fundamental to the anti-tumor response. Therefore, we next measured the effects of trilaciclib on CD19 CAR-T cell viability and proliferation. While trilaciclib had little to no effect on the viability of CD19 CAR-T cells (97%–99% viability compared with vehicle; Figure 1H), treatment significantly reduced cellular proliferation (Figure 1I), consistent with the known function of CDK4/6 in promoting cell-cycle progression.[7,11] Collectively, our findings suggest that combining a CDK4/6 inhibitor with CAR-T cell therapy may reduce CAR-T cell expansion while simultaneously enhancing CAR-T cell function via improved metabolic fitness and cytotoxic efficacy.

To determine whether these effects were specific to trilaciclib, we carried out several *in vitro* analyses using an alternative CDK4/6 inhibitor, palbociclib, as well as a CDK4-selective inhibitor, atirmociclib.[19] Similar to trilaciclib, both palbociclib and atirmociclib reduced proliferation of CAR-T cells, with a significant increase in cells arrested in G1 and a corresponding reduction in S-phase cells (Figure S1A). Induction of G1 arrest was slightly stronger with both trilaciclib and palbociclib compared with atirmociclib (99.7% vs. 96.6%). All three inhibitors enhanced CAR-T cell cytotoxic function to a similar extent, including tumor cell killing, degranulation, and cytokine production (Figures S1C and S1D). However, only trilaciclib and palbociclib, but not atirmociclib, increased the stem-cell memory T cell (Tscm) population (Figures S1B and S2A). These findings suggest that strong simultaneous inhibition of both CDK4 and CDK6 may be required for coordinated induction of stem-like features alongside enhanced effector function.

Combination CAR-T cell therapy and CDK4/6 inhibition demonstrates additive effects in RB-proficient blood cancer

To evaluate the efficacy of combination CDK4/6 inhibition and CD19 CAR-T cell therapy *in vivo*, we inoculated immunodeficient NOD SCID Gamma (NSG) mice with luciferase-expressing NALM-6 tumors and treated them with human CD19 CAR-T cells generated from healthy human donors. Starting the day of CAR-T cell transfer, mice were administered trilaciclib daily following a continuous therapy cycle of 3 days on and 4 days off treatment (Figure 2A). As transcriptional reprogramming is reported to occur in T cells in as little as 24 h of CDK4/6 inhibition,[11] a schedule of 3 days on and 4 days off was chosen with a goal to induce favorable effects on CAR-T cell phenotype, while enabling a window for CAR-T cells to expand during regular periods of treatment withdrawal. Notably, this treatment regimen was well tolerated, both as a single agent and in combination with CAR-T cell therapy, with no reduction in mouse weight or increase in systemic cytokines observed compared with vehicle-treated mice (Figures 2B and 2C). Tumor burden was tracked in mice over time by weekly luciferin injections and bioluminescence imaging (BLI) (Figure 2D). As single agents, both CAR-T cells and trilaciclib led to a marked reduction in tumor burden and a significant improvement in mouse survival, measured by the time taken for mice to reach ethical endpoints (median survival 26 and 28 days respectively versus 16 days in vehicle-treated mice; Figures 2D–2F). Tumor progression was further attenuated by the combination of trilaciclib and CAR-T cell therapy, with the combination group exhibiting a significant improvement in

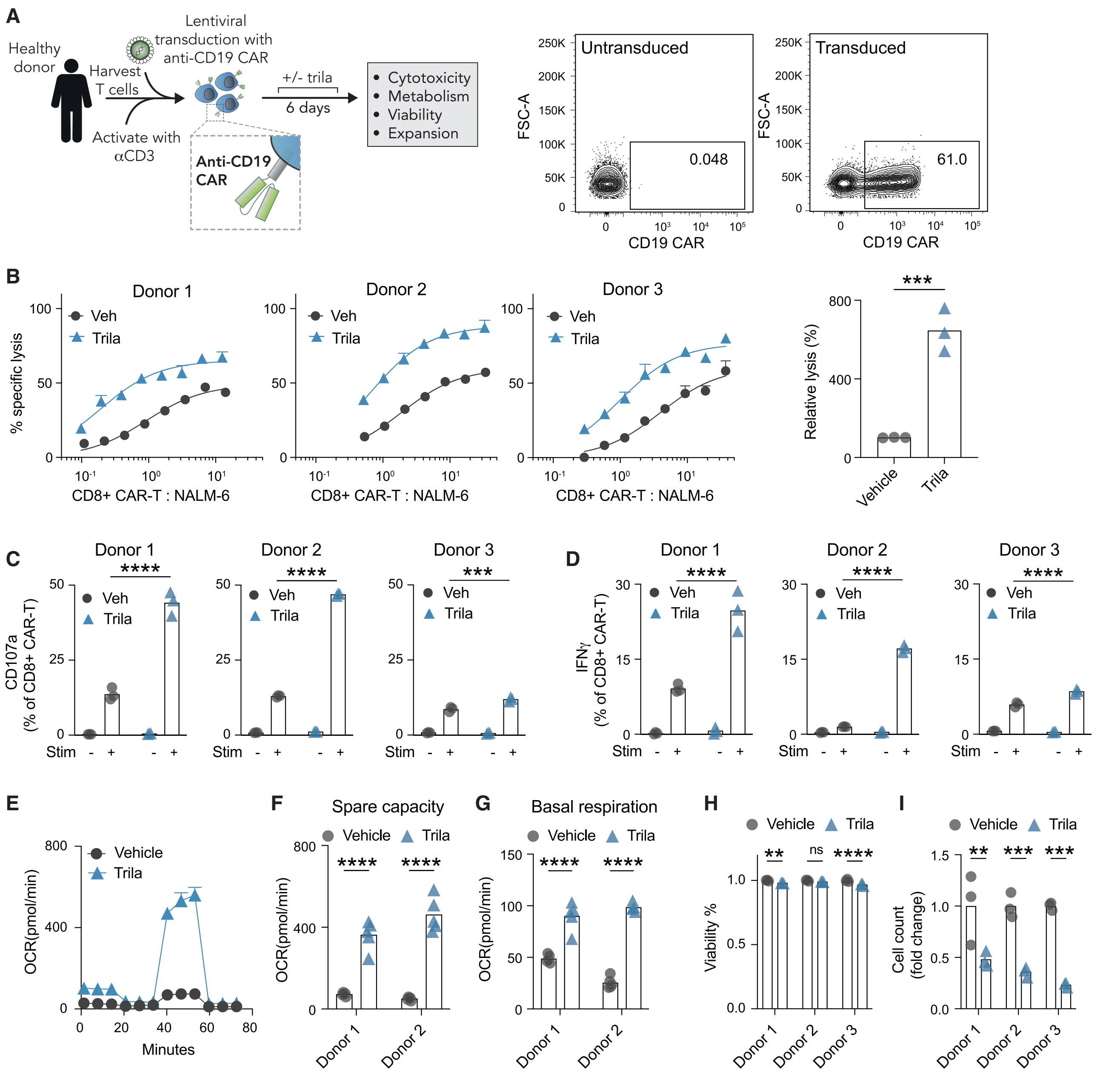

Figure 1. Trilaciclib enhances anti-tumor cytotoxicity and metabolic fitness of human CAR-T cells *in vitro*

(A) Schematic of *in vitro* CAR-T cell transduction and culture with trilaciclib. T cells were isolated from healthy donors, activated, and transduced to express a CD19-directed CAR. Transduced cells were then cultured for 6 days in trilaciclib or a vehicle control. CAR-T cells were washed to remove drug before use in assays. Plots show representative transduction efficiency measured by flow cytometry to detect the CD19 CAR. (B) CAR-T cell killing activity against tumor cell targets. CAR-T cells were co-cultured with 51Cr-labeled NALM6 tumor cells at increasing E:T ratios; E, effector (CD8+ CAR-T cells); T, target (NALM6 tumor cells). Specific lysis was measured by percent chromium release, and relative killing was calculated by normalizing vehicle control to maximum killing of 100%. Relative lysis analysis shows all donors pooled, Unpaired *t* test, $n = 3$ independent donors. (C) Degranulation (CD107a) and (D) IFNγ production by CAR-T cells co-cultured with NALM6 tumor cells *in vitro* for 4 h. Fluor-conjugated CD107a-targeting antibody and Golgi plug were added to co-cultures before staining for analysis by flow cytometry. Two-way ANOVA Sidak's multiple comparisons test, $n = 3$ across 3 independent donors. (E–G) CAR-T cell metabolic fitness measured by Seahorse XF Cell Mito Stress Test, normalized to live cell number, showing (E) mitochondrial metabolism, (F) spare respiratory capacity, and (G) basal respiration. Two-way ANOVA Sidak's multiple comparisons test, $n = 5$ across 2 independent donors. (H) CAR-T cell viability measured by flow cytometry to detect PI uptake. (I) CAR-T cell number measured by flow cytometry using count beads. Two-way ANOVA Sidak's multiple comparisons test, $n = 3$ across 3 independent donors. All error bars show ± SEM, $^{**}p < 0.01$, $^{***}p < 0.001$, $^{****}p < 0.0001$.

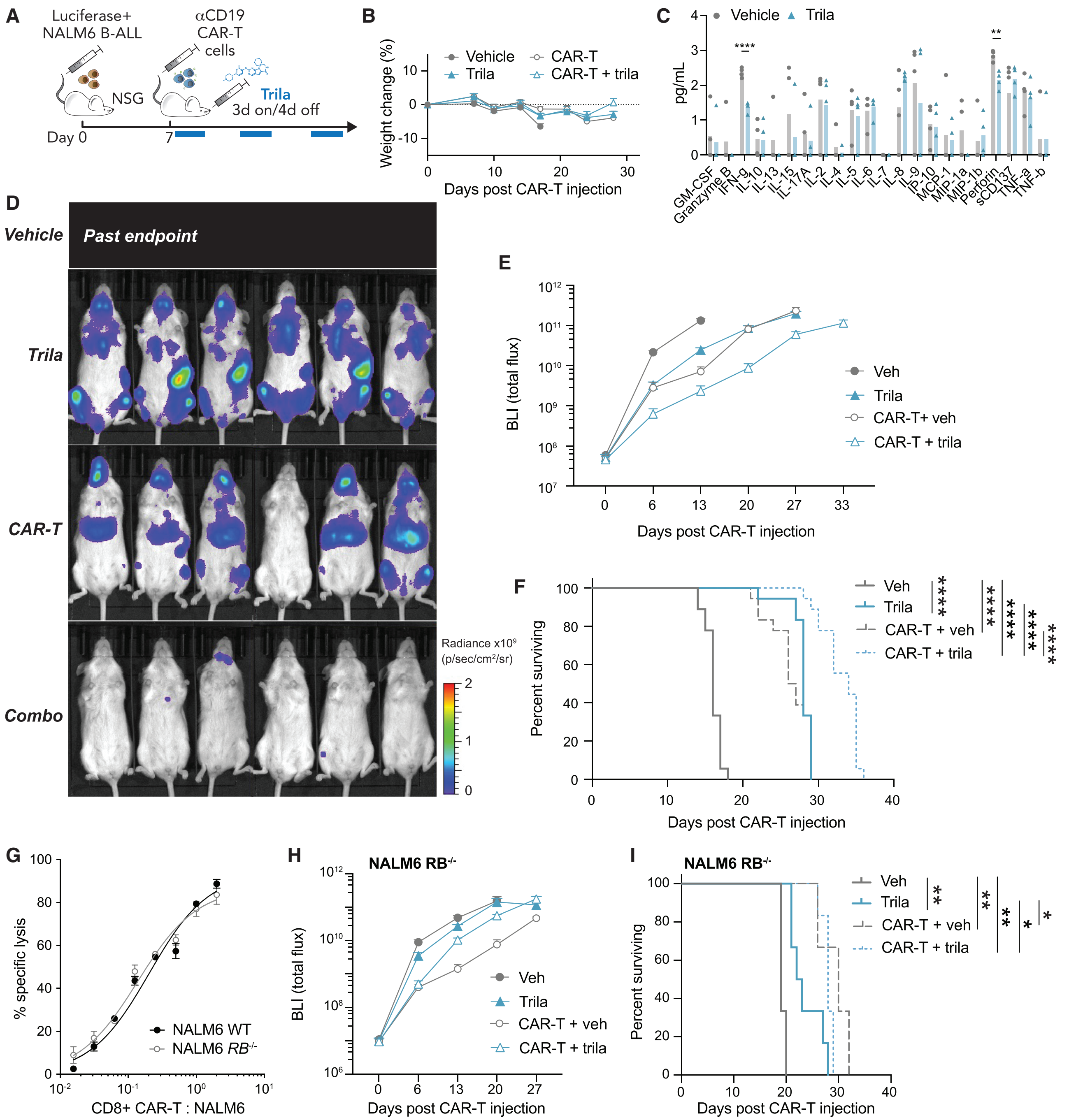

Figure 2. Combination CAR-T cell therapy and trilaciclib is efficacious in RB-proficient blood cancer

(A) Schematic of combination treatment schedule of trilaciclib and CAR-T cell therapy. NALM6-bearing immunodeficient NSG mice were given an ongoing treatment cycle of trilaciclib or a corresponding vehicle daily for 3 days, followed by 4 days without treatment, starting the day of CAR-T cell transfer and continuing until mice reached ethical endpoint. (B) Weights of mice measured over time in each treatment group. (C) Analysis of serum cytokines in mice 10 days post CAR-T cell transfer, measured using Isoplexis assay. Multiple unpaired *t* tests, $n = 4$. (D) Representative BLI images taken 13 days post CAR-T cell transfer. (E) Tumor burden over time measured by BLI. (F) Survival measured as time taken for mice to reach ethical endpoint. Survival comparisons made using log rank (Mantel-Cox) test. (D–F) Data pooled from 3 independent experiments, total $n = 18$ mice/group. (G) *In vitro* CAR-T cell efficacy against wild-type and RB-deficient NALM6-cells. CAR-T cells were co-cultured with 51Cr-labeled NALM6 tumor cells at increasing E:T ratios; E, effector (CD8+ CAR-T cells); T, target (NALM6 tumor cells). (H) Tumor burden over time measure by BLI and (I) survival in mice bearing RB-deficient NALM-6 tumors treated with the same dosing regimen shown in (A), with pooled data from 2 independent experiments, $n = 6$ mice/group, and survival comparisons were made using log rank (Mantel-Cox) test. All error bars show ± SEM, $*p < 0.05$, $**p < 0.01$, $****p < 0.0001$.

survival compared with either of the single agents (median survival 34 days; Figure 2F).

As trilaciclib was effective against NALM-6 tumors as a single agent, the direct effect of trilaciclib on the efficacy of CAR-T cell therapy remained unclear. Specifically, it was not possible to delineate the tumor response to each individual therapy from the response driven by combinational interactions. We therefore established a CDK4/6 inhibitor-resistant NALM-6 tumor cell line through CRISPR-Cas9-mediated deletion of RB, as RB is the predominant substrate of CDK4/6 and is required for cell-cycle arrest in response to CDK4/6 inhibitors.[7] Deletion of RB did not affect the susceptibility of NALM-6 cells to CD19 CAR-T cell killing *in vitro* (Figure 2G). RB-deficient NALM6 tumors were established in immunodeficient NSG mice, which were then administered CD19 CAR-T cells in combination with trilaciclib or a vehicle control. As expected, RB-deficient NALM6 tumors showed diminished sensitivity to trilaciclib but retained their sensitivity to CAR-T cell therapy. Notably, unlike NALM6 wild-type tumors, RB-deficient NALM6 tumors showed no improvement in the response to combination treatment compared with CAR-T cell therapy alone, as measured by tumor burden and mouse survival (Figures 2H and 2I). Consistent with our *in vitro* analyses, trilaciclib significantly reduced expansion of CAR-T cells in both cancer models, while we observed no significant differences in CAR-T cell expansion or differentiation, or the effects or trilaciclib on these parameters, between RB-proficient and RB-deficient tumors (Figures S3A and S3B). Collectively, these data suggest that combination CDK4/6 inhibition and CAR-T cell therapy may be an effective combination for the treatment of RB-proficient blood cancer due to an additive therapeutic effect. However, in an immunodeficient model of hematological cancer, in the absence of a tumor-intrinsic response to CDK4/6 inhibition, the combination of a CDK4/6 inhibitor and CAR-T cell therapy is no more effective than single-agent CAR-T cell therapy.

CDK4/6 inhibition enhances CAR-T cell efficacy in solid tumors

While CAR-T cell therapy has been remarkably successful in treating patients with blood cancers, efficacy in solid tumors remains poor. CDK4/6 inhibitors have been shown to promote anti-tumor immunity through a variety of immunomodulatory effects that may be specifically relevant to the solid tumor setting. We therefore examined whether combination trilaciclib and CAR-T cell therapy is effective in solid tumor mouse models. In all experiments, we utilized a continuous 3 days on/4 days off trilaciclib treatment cycle starting the day of CAR-T cell transfer. We first examined models of HER2-expressing human breast carcinoma (HCC1954) and human Lewis-Y-expressing ovarian carcinoma (OVCAR3) (Figures 3A–3H), both of which are intrinsically resistant to CDK4/6 inhibition (Figure S4). Tumors were established in immunodeficient NSG mice, and HCC1954 or OVCAR3 tumor-bearing mice were then treated with either HER2-directed CAR-T cells or Lewis-Y-directed CAR-T cells, respectively. Interestingly, while each model responded poorly to trilaciclib and CAR-T cell therapy as single agents, the combination therapy showed modest efficacy against both tumor types (Figures 3A–3H). Together, this suggested that the trilaciclib may enhance CAR-T cell efficacy against solid tumors.

Given the multifaceted immunomodulatory effects of CDK4/6 inhibitors, we next examined the efficacy of combination trilaciclib and CAR-T cell therapy in fully immunocompetent models. We first utilized our previously described transgenic mouse model of human HER2-driven breast adenocarcinoma, MMTV-rtTA/tetO-HER2.[20] In this model, tumors arise spontaneously in the mature mammary tissue of immunocompetent mice following doxycycline-mediated induction of human HER2 expression. Notably, we have previously reported that CDK4/6 inhibition promotes endogenous anti-tumor immunity in this model and synergizes with ICI,[8] making it a valuable model to look for similar combinatorial effects in the context of CAR-T cell therapy. To generate CAR-T cells for this model, T cells were isolated from healthy donor mice and engineered to express a CAR targeting human HER2 (hHER2). Following tumor establishment, MMTV-rtTA/tetO-HER2 mice were treated with hHER2-CAR-T cells in combination with trilaciclib or a vehicle control, again employing a therapy cycle of 3 days on and 4 days off (Figure 3I). Consistent with our previous studies in this model, we observed a significant survival benefit in mice treated with trilaciclib as a single agent (median survival 70 days versus 52 days for vehicle-treated mice; Figures 3J-3L). CAR-T cells as a single agent were also efficacious in this model (median survival 65 days; Figures 3J-3L). Notably, the combination of trilaciclib and CAR-T cells in this model demonstrated potent efficacy and significantly improved mouse survival compared with either of the single agents (median survival 80 days; Figures 3J-3L).

To more directly examine the effects of CDK4/6 inhibition specifically on CAR-T cell-mediated tumor control, we next examined combination effects in an immunocompetent model with poor sensitivity to trilaciclib as a single agent. To do this, we used our syngeneic mouse breast carcinoma cell line E0771-hHER2, which has been genetically engineered to express human HER2.[21–23] E0771-hHER2 tumors were established in the mammary tissue of immunocompetent C57BL/6 mice, which were then treated with mouse hHER2 CAR-T cells in combination with trilaciclib or a vehicle control (Figure 3M). While CAR-T cells and trilaciclib showed only modest activity against E0771-hHER2 tumors as single agents, the combination led to a potent reduction in tumor progression and a significant improvement in survival compared with vehicle-treated mice and mice treated with either of the single agents (median survival 39 days versus 24–28 days in other treatment groups; Figures 3N-O). Collectively, our findings suggest that combination trilaciclib and CAR-T cell therapy is an effective treatment for solid tumors even in the absence of a strong response to either individual therapy.

CDK4/6 inhibition depleted Tregs and enhances CD8+ CAR-T cell anti-tumor function

To understand how trilaciclib may be enhancing CAR-T cell activity against solid tumors, we conducted further analysis using our

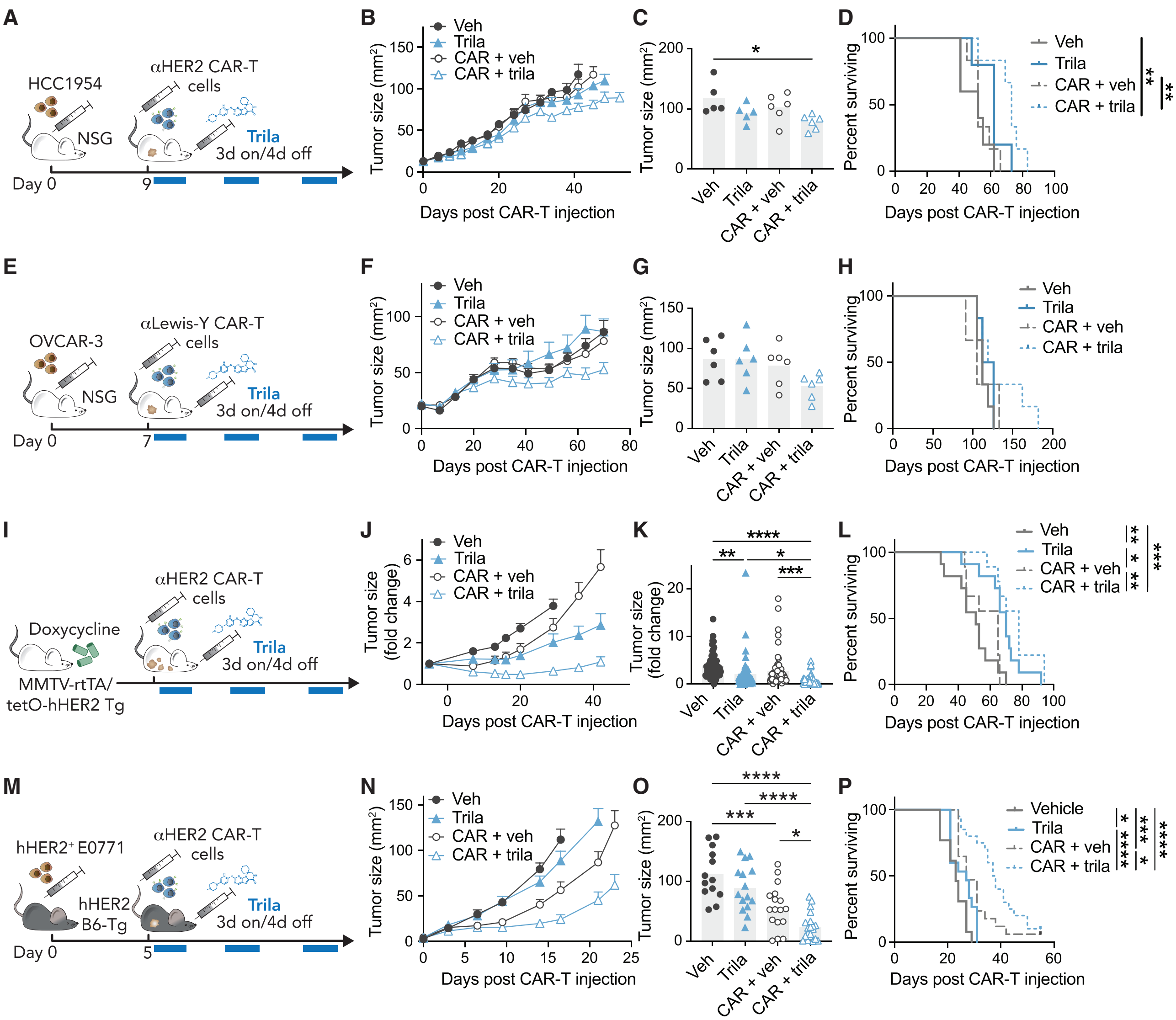

Figure 3. Trilaciclib enhances CAR-T cell efficacy in solid tumors

(A, E, I, and M) Schematic of combination treatment schedule of trilaciclib and CAR-T cell therapy in specified solid tumor models. All mice were given an ongoing treatment cycle of trilaciclib or a corresponding vehicle daily for 3 days, followed by 4 days without treatment, starting the day of CAR-T cell transfer. (A) HCC1954 tumor-bearing immunodeficient NSG mice were treated with HER2-directed human CAR-T cells. (B) Tumor growth, (C) tumor size at day 40 post CAR-T injection, and (D) survival from model (A), $n = 5$–6 mice/group, and comparisons were made using ordinary one-way ANOVA Tukey's multiple comparisons test, and survival comparisons were made using log rank (Mantel-Cox) test. (E) OVCAR-3 tumor-bearing immunodeficient NSG mice were treated with Lewis-Y-directed human CAR-T cells. (F) Tumor growth, (G) tumor size at day 70 post CAR-T injection, and (H) survival from model (E), $n = 6$ mice/group. Comparisons were made using ordinary one-way ANOVA Tukey's multiple comparisons test, and survival comparisons made using log rank (Mantel-Cox) test. (I) FVB/N-MMTV-rtTA/tetO-HER2 Tg immunocompetent mice were given a doxycycline-supplemented diet to induce tumor formation in the mammary glands. Mice developed single or multiple tumors. Tumor-bearing mice were treated with HER2-directed mouse CAR-T cells. (J and K) Fold change in the size of individual tumors from the first day of treatment is shown. (J) Tumor growth, (K) tumor size fold change at day 30 post CAR-T injection, and (L) survival from model (I), with data pooled from 2 independent experiments, $n = 58$–64 tumors/group from $n = 9$–11 mice/group, and comparisons were made using ordinary one-way ANOVA Tukey's multiple comparisons test, and survival comparisons were made using log rank (Mantel-Cox) test. (M) E0771-hHER2 tumor-bearing immunocompetent C57BL/6-hHER2 Tg mice were treated with HER2-directed mouse CAR-T cells. (N) Tumor growth, (O) tumor size at day 17 post CAR-T injection, and (P) survival from model (M), with data pooled from 2 independent experiments, $n = 13$–20 mice/group, and comparisons were made using ordinary one-way ANOVA Tukey's multiple comparisons test, and survival comparisons were made using log rank (Mantel-Cox) test. All error bars show ± SEM. $^{*}p < 0.05$, $^{**}p < 0.01$, $^{***}p < 0.001$, $^{****}p < 0.0001$.

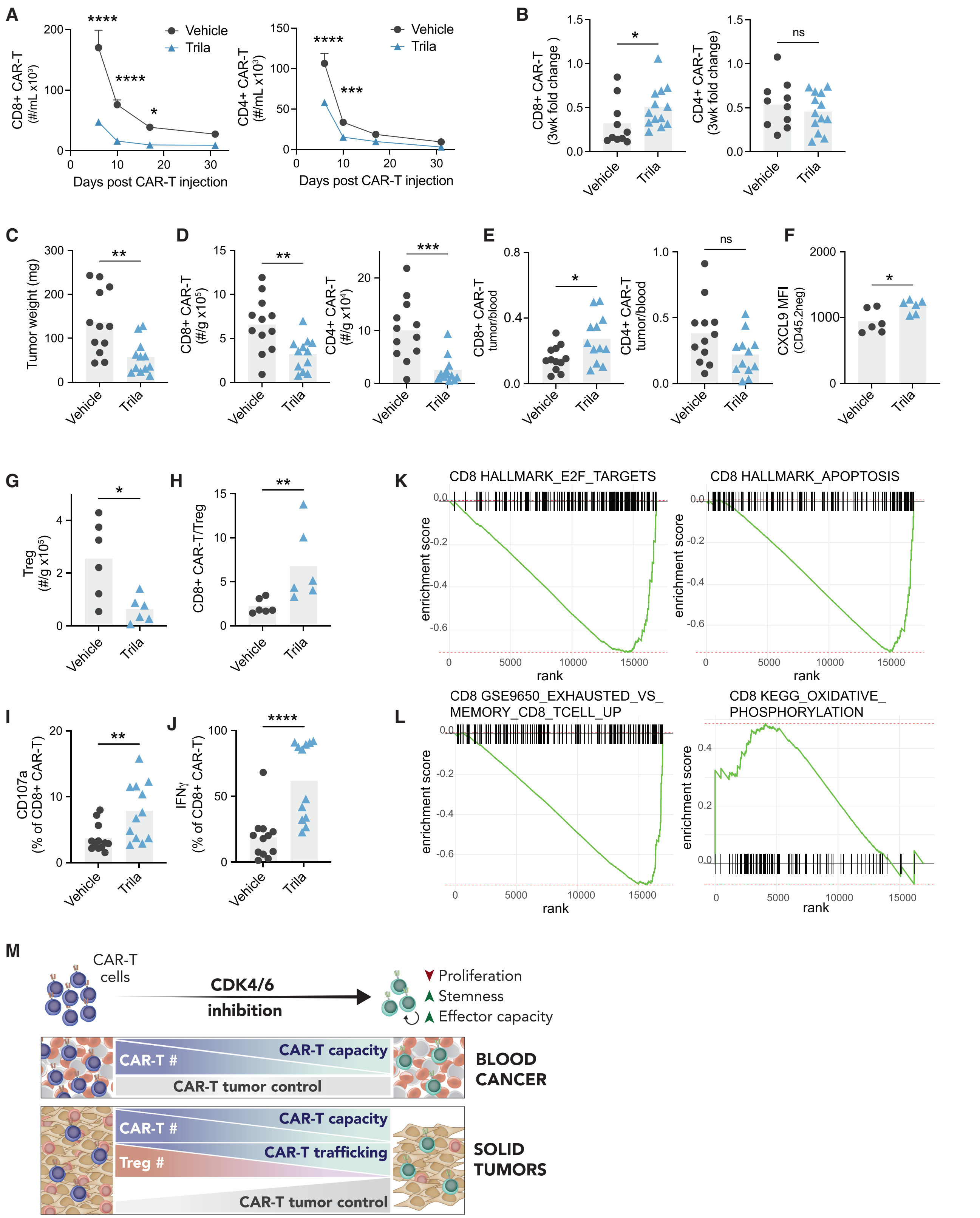

(legend on next page)

E0771-hHer2 model. Following CAR-T cell transfer into tumor-bearing mice, we collected blood to examine the numbers of circulating CAR-T cells in mice treated with trilaciclib or a vehicle control. Consistent with our *in vitro* observations that trilaciclib reduces CAR-T cell proliferation, we observed lower numbers of both CD8+ and CD4+ CAR-T cells in the blood of trilaciclib-treated mice (Figure 4A). However, when we analyzed the blood over time, we found that the number of CD8+ CAR-T cells in vehicle-treated mice decreased more rapidly than those in trilaciclib-treated mice over a 3-week period (Figures 4A and 4B). This was not observed in CD4+ CAR-T cells (Figures 4A and 4B). This suggests trilaciclib may reduce overall circulating CAR-T cell numbers via a reduction in their proliferation but simultaneously enhance the persistence of the CD8+ CAR-T cell population.

To directly address the relationship between reduced CAR-T expansion and tumor clearance, we performed a series of complementary time-resolved analyses integrating CAR-T cell numbers with tumor burden. First, we examined correlations between tumor burden and circulating CAR-T cell numbers at early and late time points in vehicle- and trilaciclib-treated mice receiving CAR-T cells. At the early time point, both groups showed a weak negative correlation, demonstrating that higher initial CAR-T cell numbers are associated with enhanced tumor control (Figure S5A). However, at the late time point, this relationship diverged, with vehicle-treated mice exhibiting a stronger negative correlation, while no correlation was observed in trilaciclib-treated mice, which instead reflected sustained tumor control despite lower numbers of CAR-T cells (Figure S5B). These findings suggest that trilaciclib enhances CAR-T efficacy through improved anti-tumor function per cell rather than numerical expansion.

To further quantify this effect, we calculated an anti-tumor effect per CAR-T cell for each individual mouse. Tumor control was determined by normalizing tumor burden in each combination-treated mouse to the average tumor burden of the corresponding drug treatment-only control group (no CAR-T) at the same time point, thereby isolating CAR-T-specific tumor control. This value was then divided by the number of CAR-T cells in the same mouse at the same time point to derive a per-cell anti-tumor potency metric over time. Using this approach, we observed a progressive increase in CAR-T potency in trilaciclib-treated mice compared with vehicle-treated mice, consistent with enhanced CAR-T cell activity despite reduced overall expansion (Figure S5C). Finally, to integrate these dynamics into a single quantitative measure of CAR-T cell potency, we compared overall CAR-T cell-mediated tumor control to overall CAR-T cell exposure over time for individual mice. Overall, CAR-T cell-mediated tumor control was calculated by subtracting the area under the curve (AUC) of tumor growth over time in mice treated with combination drug plus CAR-T cells from the average tumor AUC of the corresponding drug treatment-only group. Similarly, overall CAR-T cell exposure was determined as the AUC of CAR-T cell numbers in matched mice over time. This analysis yields a single metric that integrates cumulative tumor control with total CAR-T exposure over time, providing an overall measure of per-cell anti-tumor potency. Using this approach, we observed a significant increase in overall CAR-T potency in trilaciclib-treated mice relative to vehicle-treated controls (Figure S5D). Together, these analyses demonstrate that trilaciclib enhances CAR-T-mediated tumor clearance by increasing functional potency per cell rather than CAR-T cell number.

To determine the mechanisms by which trilaciclib enhances CAR-T cell anti-tumor potency, we harvested tumors from mice after 2 cycles of treatment (approximately 14 days after CAR-T cell transfer). Consistent with our tumor burden and survival studies, tumors isolated from trilaciclib-treated mice were significantly smaller than those harvested from vehicle-treated mice (Figure 4C). Similarly to our findings in the blood, we observed significantly fewer CD8+ and CD4+ CAR-T cells in the tumors of trilaciclib-treated mice (Figure 4D). Interestingly, when we examined the ratio of CAR-T cells in the blood versus tumor, we found a significantly higher proportion of CD8+ CAR-T cells, but not CD4+ CAR-T cells in tumors of mice treated with trilaciclib (Figure 4E), suggesting that these cells may migrate more efficiently to tumors in these mice. Indeed, we also observed higher expression of the T cell chemoattractant CXCL9 in

Figure 4. Trilaciclib depletes Tregs and enhances CD8+ CAR-T cell anti-tumor function

HER2-directed mouse CAR-T cells were transferred into immunocompetent C57BL/5 mice 5 days post mammary fat pad inoculation with the mouse breast cancer cell line E0771-hHER2, as shown in Figure 3M. Mice were treated with continuous 3 days on/4 days off trilaciclib regimen starting the day of CAR-T cell transfer. Blood was collected at regular intervals, and tumors were harvested following two cycles of 3 days on/4 days off trilaciclib. (A) Number of CAR-T cells in blood over time measured by flow cytometry. (B) Fold change in CAR-T cell numbers in blood from day 10 to day 31 post CAR-T cell transfer. (C) Weight of harvested tumors. (D) Total number of CAR-T cells in harvested tumors. (E) Ratio of the number of CAR-T cells in tumor versus blood at the time of tumor harvest. (A–E) Pooled data from 2 independent experiments, $n = 10–13$ mice/group, and comparisons were performed using Mann-Whitney nonparametric t test. (F) Chemokine expression in tumor cells (CD45.2neg). (G) Total number of endogenous regulatory T cells (Treg) in harvested tumors. (H) Ratio of tumor-infiltrating CD8+ CAR-T cells to Treg cells. (F–H) $n = 6$ mice/group, and comparisons were performed using unpaired t test. (I and J) Tumor single cell suspensions were stimulated with E0771-hHER2 cells in the presence of fluor-conjugated CD107-targeting antibody and Golgi plug for 4 h before staining for analysis by flow cytometry. Pooled data from 2 independent experiments, $n = 12$ mice/group, and comparisons were performed using Mann-Whitney nonparametric t test. (K and L) Gene set enrichment analysis of differentially expressed genes in CD8+ CAR-T cells isolated from tumors of vehicle- or trilaciclib-treated mice subjected to RNA sequencing. (M) Proposed mechanism by which CDK4/6 inhibition impacts CAR-T cell efficacy against blood cancer and solid tumors. In blood cancers, CDK4/6i-mediated induction of CAR-T stemness and effector function (capacity) is offset by reduced CAR-T cell numbers, with no overall impact of CDK4/6 inhibition on CAR-T cell-mediated tumor control. In solid tumors, CDK4/6-mediated induction of CAR-T cell stemness and effector capacity is coupled with efficient tumor trafficking and reduced immunosuppression by intratumoral regulatory T (Treg) cells, which together offset dampened CAR-T cell expansion to augment overall CAR-T cell-mediated tumor control. Error bars show ± SEM. $^{*}p < 0.05$, $^{**}p < 0.01$, $^{***}p < 0.001$, $^{****}p < 0.0001$.

the tumor cells (identified through negative expression of CD45.2) of mice treated with trilaciclib (Figure 4F). We next evaluated the endogenous tumor-infiltrating regulatory T cells (Tregs), as we and others have previously reported that CDK4/6 inhibition potently depletes this T cell subset.[8,14,24] Consistent with this, we observed a significant reduction in the number of endogenous Tregs within the tumors of mice treated with trilaciclib (Figure 4G). Notably, despite trilaciclib leading to lower numbers of CAR-T cells, this corresponding reduction in Tregs translated to a significantly higher CD8+ CAR-T cell to Treg ratio in these tumors (Figure 4H). We did not observe any significant effects on the number of myeloid populations within the tumor microenvironment following trilaciclib treatment, including total CD11b+ cells, dendritic cells, macrophages, monocytic myeloid-derived suppressor cells (MDSCs), and polymorphonuclear MDSCs (Figures S1B and S6).

As our findings suggested that trilaciclib may be enhancing the CD8+ CAR-T cell response, we further evaluated this population using *ex vivo* stimulation assays to examine CAR-T cell cytotoxic function and RNA sequencing to evaluate CAR-T cell phenotype. Following *ex vivo* stimulation with E0771-hHer2 tumor cells, CD8+ CAR-T cells from the tumors of mice treated with trilaciclib exhibited significantly higher levels of degranulation (Figure 4I) and IFNγ production (Figure 4J), indicating these cells have greater anti-tumor cytotoxic activity. Examination of differential transcripts by gene set enrichment analysis showed a downregulation of E2F targets and apoptosis genes in CD8+ CAR-T cells from the tumors of trilaciclib-treated mice (Figure 4K). This was consistent with our findings that trilaciclib reduces proliferation while enhancing survival of these cells. We also identified enrichment of genes signatures associated with reduced exhaustion and increased oxidative phosphorylation (Figure 4L), indicative of memory differentiation and cell longevity.

Collectively, our data suggest that trilaciclib may enhance CD8+ CAR-T cell anti-tumor immunity through multifaceted mechanisms, including potent Treg depletion within solid tumors, and efficient recruitment of functionally superior CD8+ CAR-T cells into the tumor microenvironment (Figure 4M).

DISCUSSION

In this study, we investigated the potential of combining the CDK4/6 inhibitor trilaciclib with CAR-T cell therapy by exploring this combination across a variety of preclinical tumor models. Numerous studies have reported potent induction of endogenous anti-tumor immunity in response to CDK4/6 inhibition,[8,9,11–14,24] and it logically follows that these inhibitors may also enhance exogenous cellular immunity. However, our study demonstrates that the utility of a CDK4/6 inhibitor as adjuvant to cellular immunotherapy is complex and context dependent. Notably, this study establishes CDK4/6 inhibition as an adjuvant strategy for CAR-T therapy, with the potential to create a net therapeutic benefit despite reduced CAR-T expansion. Importantly, we identify contextual determinants of therapeutic benefit, including tumor RB dependence in hematological cancers, and immunosuppression in solid tumors. Together, these findings shift CDK4/6 inhibitors from being considered primarily tumor-cytostatic or ICI adjuvants to being microenvironment-modulating agents that can improve CAR-T performance in settings where CAR-T may fail clinically.

The idea that CDK4/6 inhibitors may enhance exogenous immunity was first indicated by our previous work, which demonstrated that preconditioning CAR-T cells with a CDK4/6 inhibitor *in vitro* during the manufacturing protocol significantly enhances their persistence and anti-tumor function when transferred into mice.[11] While this provided clear evidence that CDK4/6 inhibition augments CAR-T cell longevity and function at the cellular level, normalization of cell numbers across those studies meant the functional consequence of reduced CAR-T cell expansion in response to CDK4/6 inhibition was not captured. It is certainly still feasible that CDK4/6 inhibitors could serve as a preconditioning tool to improve the phenotype of CAR-T cells prior to CAR-T cell infusion. However, this would need to be further explored and compared with alternative culture strategies aimed at enhancing CAR-T cell phenotype, such as the addition of IL-15 or AKT inhibitors to T cell cultures[23,25] or shortening the *in vitro* culture period.[26] Indeed, preconditioning CAR-T cell media with IL-15 overcomes the disadvantage imposed by CDK4/6 inhibitors of reducing CAR-T cell expansion. However, while these alternative approaches primarily act cell-intrinsically to enhance CAR-T cell phenotype prior to CAR-T cell transfer, they do not directly address immunosuppressive features of the tumor microenvironment. In contrast, the major advantage of CDK4/6 inhibitors over these other approaches for enhancing CAR-T cell therapy more likely stems from their multifaceted immunomodulatory activity when used systemically *in vivo*, rather than their sole direct effects on CAR-T cell phenotype. This is indeed evidenced by our findings that CDK4/6 inhibition is most effective in enhancing the CAR-T cell response against solid tumors in the presence of an endogenous immune system. Notably, this response was supported via multiple mechanisms that align with previous reports on the immunomodulatory effects of CDK4/6 inhibitors, including depletion of Tregs[8,11,14] and enhanced T cell persistence, cytotoxicity, and trafficking.[8,11–13] Furthermore, our transcriptomic data support a model in which CDK4/6 inhibition suppresses E2F-dependent proliferation programs while favoring survival and oxidative metabolism, consistent with a shift toward a memory-like state that can sustain cytotoxic function within nutrient- and oxygen-limited tumors. While we did not directly interrogate downstream signaling intermediates, our data are consistent with coordinated changes in cell-cycle control, metabolic wiring, and chemokine-guided trafficking that together synergize to increase per-cell CAR-T cell anti-tumor potency.

While CDK4/6 inhibitors enhance CAR-T cell function at the individual cell level, a major question remains as to what degree this increase in function compensates for a simultaneous reduction in expansion. Regarding the net anti-tumor potential of the resulting CAR-T cell population, the answer is not simple when considering

an *in vivo* environment that is highly dynamic, both spatially and temporally. For example, although CDK4/6 inhibitors reduce overall numbers of cytotoxic CD8+ CAR-T cells in mice, a higher proportion of these are drawn into a tumor microenvironment that is less immunosuppressive. Hence, these cells gain an advantage not only in terms of function but also location and autonomy. Furthermore, while CDK4/6 inhibition reduces overall CAR-T cell numbers in the short term, this is partly offset over time due to the ability of these cells to survive and persist for long periods. It is certainly possible that over a longer enough period, such as that which would be observed in humans compared with mice, a reduction in CAR-T cell expansion following CDK4/6 inhibition may be fully offset by improved CAR-T cell survival in the long term. In fact, in the context of clinical therapy, slowing down and prolonging the CAR-T cell response could prove to be an advantageous strategy to mitigate toxicities associated with rapid CAR-T cell expansion and cytokine release, which present a major challenge in the management of patients receiving CAR-T cell therapy.[27] Consistent with this, we observed no evidence of increased systemic toxicity in mice receiving combination treatment, as assessed by body weight and serum cytokine levels, despite CDK4/6i enhancing individual CAR-T cell effector cytokine secretion. Hence, even in settings where CDK4/6 inhibition shows no net improvement in the overall anti-cancer activity of CAR-T cells, it may still prove to be a valuable strategy for managing acute toxicities without negatively impacting therapeutic efficacy. Notably, while CDK4/6 inhibitors have been extensively evaluated clinically as monotherapies or in combination with chemotherapy or immune checkpoint blockade, their use alongside CAR-T cell therapy remains clinically unexplored, highlighting both the translational potential and the need for careful safety evaluation.

Several limitations of this study should be acknowledged. First, these findings are based on preclinical mouse models, including immunodeficient systems, which will not fully recapitulate human tumor immune dynamics. Second, mechanistic conclusions are largely inferred from phenotypic, functional, and transcriptional analyses. Finally, dose and timing of CDK4/6 inhibition relative to CAR-T cell infusion were not exhaustively explored and may significantly influence therapeutic outcome. Future studies may seek to evaluate additional treatment regimens to more optimally balance the favorable effects of CDK4/6 inhibition on CAR-T cell activity with the corresponding reduction in CAR-T cell expansion.

In conclusion, this study identifies CDK4/6 inhibition as a context-dependent adjuvant strategy for CAR-T cell therapy, with particular relevance to solid tumors characterized by immunosuppressive microenvironments. These findings support future investigation into optimized dosing and scheduling strategies, as well as biomarker-guided patient selection, such as tumor RB status in hematological malignancies or baseline immune suppression in solid tumors. More broadly, this work provides a framework for leveraging clinically approved CDK4/6 inhibitors to enhance CAR-T cell efficacy by modulating the tumor microenvironment and T cell functional state, thereby extending the therapeutic potential of CAR-T cell therapy beyond its current indications.

MATERIALS AND METHODS

Pharmacological inhibitors

Trilaciclib (2'-((5-(4-methylpiperazin-1-yl)pyridin-2-yl)amino)-7′, 8′-dihydro-6′H-spiro[cycloh exane-1,9′-pyrazino[1′,2':1,5]pyrrolo [2,3-d]pyrimidin]-6′-one) was kindly provided by Pharmacosmos (Denmark). Trilaciclib was prepared in 50 mM pH 4.5 citrate buffer (Sigma-Aldrich) for *in vivo* applications or in DMSO for *in vitro* expe riments. Palbociclib (6-Acetyl-8-cyclopentyl-5-methyl-2-[[5-(1-pipe razinyl)-2-pyridinyl]amino]pyrido[3-d]pyrimidin-7(8H)-one, #HY-50767 MedChemExpress) was prepared at 10 mM in DMSO for *in vitro* experiments. Atirmociclib ((3S,4R)-4-[[5-chloro-4-[7-fluo ro-2-(2-hydroxypropan-2-yl)-3-propan-2-ylbenzimidazol-5-yl]pyri midin-2-yl]amino]oxan-3-ol, #E1495 Selleckchem) was prepared at 10 mM in DMSO for *in vitro* experiments.

Dose response assay

MCF7, OVCAR3, and HCC1954 cells were plated in 96-well plates and treated with a ten-point dose concentration from 15.625 nmol/L to 4,000 nmol/L trilaciclib for 72 h. Plates were fixed with 4% PFA and stained with DAPI (5 mg/mL, Invitrogen, cat. D1306). Plates were imaged on the CellInsight CX7 LZR Pro, and cells were counted using the Thermo Fisher Scientific HCS Studio Cell Analysis Software.

BrdU cell-cycle assay

1 hour prior to analysis, 10 μmol/L BrdU was applied to the cells. Cells were stained using the Near-IR Fixable Viability Stain (Invitrogen, L34976) for 20 min at room temperature. Cells were fixed and permeabilized using FoxP3/Transcription Factor Staining Buffer Set (eBioscience, 00–5523–00). DNA was denatured using 2 N HCl - +0.5% (v/v) Triton X-100 for 30 min. The acid was neutralized using 0.1 mol/L Na2B4O7.10H2O (pH 8.5) followed by 0.5% BSA in PBS. Cells were then stained with BrdU antibody (clone 3D4, BioLegend, 364108) for 1 h. Prior to acquisition on the BD A3 Symphony, DNA was stained using FxCycle Violet (Invitrogen, Cat. F10347).

Human and mouse T cell isolation and CAR-T cell generation

Human peripheral blood mononuclear cells (PBMCs) were isolated from buffy coats of healthy donors and virally transduced with anti-human CD19, Lewis-Y, or HER2 CARs. Specifically, PBMCs were resuspended in T cell media (RPMI 1640 containing 20 mM HEPES, 10% FBS, 1% GlutaMAX, 1 mM Sodium Pyruvate, 1 mM MEM Non-Essential Amino Acids, 0.1% 2-mercaptoethanol and Antibiotic-Antimitotic), stimulated with anti-human CD3 (OKT3 30 ng/mL, Ortho Biotech), IL-2 (100U/mL), and IL-7 (5ng/mL) and cultured at 37°C at 5% CO_2. Mouse splenocytes from C57BL/6 mice were resuspended to a density of 1×10^6 cells/mL in T cell media and activated with anti-CD3 (1g/mL) plus anti-CD28 (2 μg/mL) in the presence of IL-2 (100 IU/mL) and IL-7 (2 ng/mL), followed by retroviral transduction with an anti-human HER2 CAR. On days 2 and 3 post activation (for human) or days 1 and 2 post activation (for mouse), viral supernatant was spun at

2000 × *g* for 1 h onto Retronectin-coated non-tissue culture 6-well plates (Retronectin from Takara Bio, Otsu, Japan, plates from Becton Dickinson, Franklin Lakes, NJ, USA), before adding activated T cells at a density of 1×10^6 cells/mL. After 4 h, viral medium was replaced with T cell medium supplemented with IL-2 (100 U/mL) and IL-7 (5 ng/mL). Transduced T cells were expanded for 7 days and then transferred into mice for *in vivo* studies, or cultured for a further 6 days in the presence of 1 μM trilaciclib, 1 μM palbociclib, 1 μM atirmociclib, or corresponding vehicle for *in vitro* assays.

CAR design and virus generation

CD19 CAR virus was produced by transient transfection of HEK293T cells with the following plasmids (based on 3rd generation 4-plasmid helper packaging lentiviral system): (1) HIV Rev encoding helper plasmid, (2) HIV Gag/Pol encoding helper plasmid, (3) VSV-G encoding plasmid, and (4) transfer plasmid containing the transgene. The transgene encoded a CD8 signal peptide, followed by the CD19-targeting single-chain variable fragment (scFv) derived from the FMC63 antibody linked to a CD8 hinge and transmembrane domain, 4-1BB intracellular domain, and CD3-zeta intracellular domain. Lewis-Y CAR virus was produced using the PG13 retroviral producer cell line. DNA encoding Lewis-Y CAR was generated with standard molecular biology techniques as described previously.[11] Briefly, the scFv was generated from the humanized monoclonal antibody Hu3S193,[28,29] and a truncated human CD34 was inserted as an extracellular domain.[30] HER2 CAR virus was produced using the GP86 retroviral producer cell line, and the CAR consists of an extracellular scFv targeting human HER2, fused to the transmembrane domains of CD28 and CD3, as previously described.[21,31–33]

In vivo studies

Animal work was conducted with approval from the Peter MacCallum Animal Experimentation Ethics Committee in accordance with the NHMRC Australian code for the use of animals for research purposes 8th edition (2013). C57BL/6 mice were purchased from Walter Eliza Hall Institute. C57BL/6-hHER2 Tg mice and NSG mice were bred in-house. FVB/N-Tg(MMTV-rtTA/tetO-HER2) mice were bred in-house and at 6 weeks of age given a diet containing 2500 mg/kg doxycycline (SF19–128; Specialty Feeds) for 8–12 weeks to induce tumor establishment in mammary glands. Each mouse developed single or multiple tumors, and each individual tumor was monitored for *in vivo* analyses. All transplantable tumor cell lines were resuspended in PBS for injection. For blood cancer models, 0.5×10^6 wild-type or 1×10^6 RB-deficient NALM6 luciferase-expressing tumor cells were transferred into NSG mice via tail vein injection. For solid tumor models, 5×10^6 OVCAR-3 or HCC1954 were injected subcutaneously on the right flank of NSG mice, and 0.1×10^6 E0771-hHER2 cells were injected into the mammary fat pad of C57BL/6 mice. All mice were randomized prior to treatment based on total tumor burden. To minimize potential confounders, mice were allocated to new cages following randomization, and all groups were monitored and treated on the same day. Mice within the same treatment group were housed in the same cage to minimize the chance for treatment errors and eliminate exposure of alternative treatments through shared bedding. Mice that failed to establish tumors were excluded from the experiment. Following randomization, no mice were excluded from the analysis. Group allocation, treatment administration, and outcome measurements were performed by one researcher who was aware of group assignments. Data analysis was conducted by a second researcher who was not involved in the experiments and did not have access to group allocation during analysis. To ensure adequate statistical power, a minimum of 6 mice per group were used for all experiments, and experiments were conducted independently at least twice. Blood cancer burden was measured weekly by intraperitoneal injection of anesthetized mice with 150 mg/kg D-Luciferin followed by BLI using an IVIS Lumina XMRS, with analysis performed using Living Image software, and survival was measured as the time taken for mice to reach pre-determined ethical endpoints. Solid tumor burden was measured using calipers, and survival was measured as the time taken for tumors to reach ethical endpoint of 200 mm^2. Following tumor establishment, mice were randomized and treated with 100 mg/kg trilaciclib or the corresponding vehicle (50 mM pH4.5 citrate buffer; Sigma-Aldrich) daily by oral gavage, on a continuous cycle of 3 days on and 4 days off treatment with or without a single injection of CAR-T cells. In all experiments, CAR-T cells were resuspended in PBS prior to transfer into mice via tail vein injection. 1×10^6 or 10×10^6 CAR-T cells were transferred per mouse for blood cancers or solid tumor models, respectively. For the transplantable solid tumor models HCC1954, OVCAR-3, and E0771-hHER2, mice in all treatment groups were lymphodepleted using 5 Gy gamma irradiation immediately prior to CAR-T cell transfer and were administered 50,000 IU IL-2 daily for 4 days starting on the day of CAR-T cell transfer. All mice within these models, including those not receiving CAR-T cells, underwent identical irradiation and IL-2 administration protocols to control for any potential anti-tumor effects of these interventions.

Tumor cell lines

HCC1954, OVCAR-3, and NALM-6 were purchased from ATCC, and E0771-hHER2 expressing human HER2, as previously described, was provided by Professor Paul Beavis.[21] Human cell lines (HCC1954, OVCAR-3, NALM-6) were cultured in RPMI 1640 plus 10% FCS and cultured at 37°C in 5% CO_2. Mouse E0771-hHER2 cells were cultured in DMEM plus 10% FCS and cultured at 37°C in 10% CO_2. Luciferase-expressing NALM-6 cells were generated by transduction of NALM-6 parental cells with lentivirus encoding enhanced green fluorescent protein (eGFP) and click beetle green luciferase (CBG) in a bicistronic plasmid and sorted three times using fluorescence-activated cell sorting (FACS) to obtain a pure GFP-positive cell suspension. RB-deficient NALM-6 cells were generated by nucleofection (4D-Nucleofector; Lonza Bioscience) with Alt-R sgRNA (GCUCUUUCUCUGACAUGAUC) and purified Alt-R SpCas9 Nuclease (Integrated DNA Technologies). Single cell colonies of RB-deficient NALM-6 cells were selected via FACS sorting.

Ex vivo tissue processing and *ex vivo* stimulation

Blood was collected via tail vein into 4% citrate buffer, diluted with an equal volume of DMEM plus 2% FCS, layered onto a Lymphocyte Separation Medium (MP Biomedicals) density gradient, and centrifuged for 15 min at 930 × *g* to isolate mononuclear cells. Tumors were weighed and then enzymatically digested in DMEM containing Collagenase IV (1.6 mg/mL) and DNase (2 U/mL) at 37°C with agitation for 45 min. The resulting suspension was passed through a 70-μm filter to remove debris. For *ex vivo* simulations (used for CXCL9, CD107a and IFNγ analyses), processed tumor samples were co-cultured with E0771-hHer2 cells in the presence of fluor-conjugated CD107-targeting antibody and Golgi plug for 4 h. All samples were plated with equal numbers of count beads (Beckman Coulter Flow-Count Fluorospheres 7547053) before staining for analysis by flow cytometry.

Flow cytometry

Anti-mouse antibodies used were as follows: CD3 (17A2), TCRB (H57-597), CD8α (53–6.7), CD4 (GK1.5), CD45.1 (A20), CD45.2 (104), CD107a (1D4B), IFN-γ (XMG1.2), FOXP3 (206D), MHC-II (2G9), CD11b (M1/70), CD11c (N418), Ly6C (HK1.4), and F4/80 (BM8). CD19 CAR was detected using a PE-labelled anti-FMC63 anti-idiotype antibody (FM3-HPY53, Acor Biosystems). Cells were fixed and permeabilized for intracellular staining (CD107a, IFNγ, and FOXP3) using the Foxp3/Transcription Factor Staining Buffer Set following the manufacturers protocol (eBioscience 00-5523-00). Analysis was performed on BD LSR Fortessa X-20 or BD FACSymphony flow cytometer (BD Biosciences, North Ryde, New South Wales, Australia). Data were analyzed using FlowJo software.

CAR-T cell cytotoxicity assays

CAR-T cell cytotoxicity was assessed using a standard chromium release assay, following established protocols.[34,35] Specific killing was calculated using the formula: (Sample ^{51}Cr release − Spontaneous ^{51}Cr release)/(Total ^{51}Cr release − Spontaneous ^{51}Cr release) × 100. All assays were conducted in triplicate wells. To generate relative killing (fold) graphs, the killing efficiency at the E:T ratio corresponding to 50% of the maximal killing in the least cytotoxic condition was analyzed using Michaelis-Menten kinetics, as previously described.[35] For cytokine analysis, CAR-T cells were co-cultured with E0771-hHer2 cells in the presence of fluor-conjugated CD107-targeting antibody and Golgi plug for 4 h before staining for analysis by flow cytometry.

Proliferation and cell death assays

Absolute cell numbers and live cell percentages were determined by adding equal volumes of vehicle- and trilaciclib-treated CAR-T cell cultures to equal numbers of count beads (Beckman Coulter Flow-Count Fluorospheres 7547053) and 1 μM propidium iodide (PI). Flow cytometry was used to measure viability by PI uptake and cell number by count beads.

Metabolic assays

Mitochondrial function was assessed using the Seahorse XF Cell Mito Stress Test, as previously described.[18] Briefly, cells were seeded as 5 replicates per condition in a Seahorse XF cell culture microplate at a density of 0.2 × 10^6 cells/well in Seahorse XF Assay Medium (pH 7.4), supplemented with 25 mM glucose, 1 mM sodium pyruvate, and 2 mM L-glutamine. Cells were incubated in a non-CO_2 incubator for 1 h prior to the assay to equilibrate conditions. Oxygen consumption rate and extracellular acidification rate were measured in real time using the Seahorse XF analyzer. The following mitochondrial stressors were sequentially injected: 1 μM oligomycin, 1.5 μM FCCP, and 100 nM rotenone plus 1 μM antimycin A. Cell numbers were recounted immediately after the assay, and measurements were normalized to live cell number across wells.

RNA sequencing

Sequencing libraries were generated using the NEBNext Ultra II Directional RNA Library Prep Kit for Illumina. Libraries were sequenced on the Illumina NextSeq 500 platform to obtain 75-bp paired-end reads. Sequencing data were demultiplexed using Bcl2fastq (v2.17.1.14) to generate FASTQ files, which were subjected to quality control using FASTQC (v0.11.5). Sequencing reads were adapter-trimmed and filtered for quality using BBmap (v38.96), using the bbduk.sh script. These reads were aligned to the mouse (mm10) reference genome and tallied using Subread (v2.0.0). Differential gene expression analysis was conducted with DESeq2 (v1.36.0) with comparison of trilaciclib (n = 3) versus Citrate (n = 3) buffer groups excluding one replicate from the trilaciclib group due to technical issues resulting in an outlier. The package msigdbr (v7.5.1) was used to load gene sets from Hallmarks, Kegg, and Immunesig. The fgsea bioconductor package (v1.2.0) was used to conduct gene set enrichment analysis and generate barcode plots.

Statistical analysis

All statistical analyses were performed using GraphPad Prism version 10.3.1. Statistical tests were selected based on the experimental design, number of groups, and data distribution and are specified in the corresponding figure legends. For comparisons between two groups, unpaired two-tailed Student's t tests were used for normally distributed data or Mann-Whitney tests for nonparametric data. For experiments involving multiple groups or variables, one-way or two-way ANOVA was performed as appropriate, followed by Sidak's or Tukey's multiple-comparisons tests. Survival analyses were conducted using the log rank (Mantel-Cox) test. Where multiple comparisons were performed, correction for multiple testing was applied as indicated in the figure legends. Data are presented as mean ± SEM. Biological replicates are defined as independent human donors or individual mice, as specified for each experiment. Mouse sample sizes were determined based on prior experience with these established models, expected effect sizes, and variability observed in historical datasets, consistent with standard practice in preclinical CAR-T studies, rather than formal a priori power calculations. In some cases, final group sizes reflect pooling of animals across independent experiments with identical experimental conditions, to maximize statistical power and avoid exclusion of data, while adhering to ethical principles for animal use. *In vivo* experiments were performed independently at least twice using separate

experimental cohorts, as indicated in the figure legends. Where data were pooled across experiments, identical experimental conditions were used, and consistency of outcomes was confirmed prior to pooling. Biological replicates are defined as individual mice. For *in vitro* experiments involving human CAR-T cells, biological replicates represent independent donors, and all donor replicates are shown. For *in vivo* experiments involving human CAR-T cells, the same donor was used for individual experiments, with biological replicates represented by individual mice. No systematic batch effects were observed across independent *in vivo* experiments. A p value < 0.05 was considered statistically significant.

DATA AND CODE AVAILABILITY

The data supporting this study have been submitted to the Gene Expression Omnibus (GEO): GSE326798 and will be made publicly available upon publication. Additional data are available from the corresponding author upon reasonable request.

ACKNOWLEDGMENTS

We acknowledge the following Peter MacCallum Cancer Centre Research Division cores: The Victorian Center for Functional Genomics, Molecular Genomics, Flow Cytometry Facility, and Animal Facility. This work was supported by the National Breast Cancer Foundation (IIRS-18-151, J.O) and the CASS Foundation (E.J.L). All animal studies were performed in accordance with the NHMRC Australian Code for the Care and Use of Animals for Scientific Purposes 8th edition (2013) and with approval from the Peter MacCallum Cancer Centre Animal Experimentation Ethics Committee (ethics approvals E548, E638, and 2023-17).

AUTHOR CONTRIBUTIONS

E.J.L, J.O., and S.G. performed study concept and design; E.J.L, K-H.L., K.C., J.O., and S.G. performed development of methodology and writing, review, and revision of the paper; E.J.L., J.N., K-H.L., K.C., K.M.R., I.M., S.J., J.L., and CW.C. provided acquisition, analysis, and interpretation of data and statistical analysis; E.J.L., K.B., S.G., P.A.B., S.G., and J.O. provided technical and material support.

DECLARATION OF INTERESTS

The authors declare they have no competing financial interests. All authors read and approved the final manuscript and consent to publication.

SUPPLEMENTAL INFORMATION

Supplemental information can be found online at https://doi.org/10.1016/j.ymthe.2026.03.024.

REFERENCES

1. Sadelain, M., Rivière, I., and Riddell, S. (2017). Therapeutic T cell engineering. Nature *545*, 423–431. https://doi.org/10.1038/nature22395.
2. Albelda, S.M. (2024). CAR T cell therapy for patients with solid tumours: key lessons to learn and unlearn. Nat. Rev. Clin. Oncol. *21*, 47–66. https://doi.org/10.1038/s41571-023-00832-4.
3. Sterner, R.C., and Sterner, R.M. (2021). CAR-T cell therapy: current limitations and potential strategies. Blood Cancer J. *11*, 69. https://doi.org/10.1038/s41408-021-00459-7.
4. Shah, N.N., Lee, D.W., Yates, B., Yuan, C.M., Shalabi, H., Martin, S., Wolters, P.L., Steinberg, S.M., Baker, E.H., Delbrook, C.P., et al. (2021). Long-Term Follow-Up of CD19-CAR T-Cell Therapy in Children and Young Adults With B-ALL. J. Clin. Oncol. *39*, 1650–1659. https://doi.org/10.1200/JCO.20.02262.
5. Schultz, L. (2020). Chimeric Antigen Receptor T Cell Therapy for Pediatric B-ALL: Narrowing the Gap Between Early and Long-Term Outcomes. Front. Immunol. *11*, 1985. https://doi.org/10.3389/fimmu.2020.01985.
6. Robert, C., Ribas, A., Schachter, J., Arance, A., Grob, J.J., Mortier, L., Daud, A., Carlino, M.S., McNeil, C.M., Lotem, M., et al. (2019). Pembrolizumab versus ipilimumab in advanced melanoma (KEYNOTE-006): post-hoc 5-year results from an open-label, multicentre, randomised, controlled, phase 3 study. Lancet Oncol. *20*, 1239–1251. https://doi.org/10.1016/S1470-2045(19)30388-2.
7. Matsushime, H., Ewen, M.E., Strom, D.K., Kato, J.Y., Hanks, S.K., Roussel, M.F., and Sherr, C.J. (1992). Identification and properties of an atypical catalytic subunit (p34PSK-J3/cdk4) for mammalian D type G1 cyclins. Cell *71*, 323–334.
8. Goel, S., DeCristo, M.J., Watt, A.C., BrinJones, H., Sceneay, J., Li, B.B., Khan, N., Ubellacker, J.M., Xie, S., Metzger-Filho, O., et al. (2017). CDK4/6 inhibition triggers anti-tumour immunity. Nature *548*, 471–475. https://doi.org/10.1038/nature23465.
9. Schaer, D.A., Beckmann, R.P., Dempsey, J.A., Huber, L., Forest, A., Amaladas, N., Li, Y., Wang, Y.C., Rasmussen, E.R., Chin, D., et al. (2018). The CDK4/6 Inhibitor Abemaciclib Induces a T Cell Inflamed Tumor Microenvironment and Enhances the Efficacy of PD-L1 Checkpoint Blockade. Cell Rep. *22*, 2978–2994. https://doi.org/10.1016/j.celrep.2018.02.053.
10. Teo, Z.L., Versaci, S., Dushyanthen, S., Caramia, F., Savas, P., Mintoff, C.P., Zethoven, M., Virassamy, B., Luen, S.J., McArthur, G.A., et al. (2017). Combined CDK4/6 and PI3Kalpha Inhibition Is Synergistic and Immunogenic in Triple-Negative Breast Cancer. Cancer Res. *77*, 6340–6352. https://doi.org/10.1158/0008-5472.CAN-17-2210.
11. Lelliott, E.J., Kong, I.Y., Zethoven, M., Ramsbottom, K.M., Martelotto, L.G., Meyran, D., Zhu, J.J., Costacurta, M., Kirby, L., Sandow, J.J., et al. (2021). CDK4/6 inhibition promotes anti-tumor immunity through the induction of T cell memory. Cancer Discov. *11*, 2582–2601. https://doi.org/10.1158/2159-8290.CD-20-1554.
12. Heckler, M., Ali, L.R., Clancy-Thompson, E., Qiang, L., Ventre, K.S., Lenehan, P., Roehle, K., Luoma, A., Boelaars, K., Peters, V., et al. (2021). Inhibition of CDK4/6 promotes CD8 T-cell memory formation. Cancer Discov. *11*, 2564–2581. https://doi.org/10.1158/2159-8290.CD-20-1540.
13. Deng, J., Wang, E.S., Jenkins, R.W., Li, S., Dries, R., Yates, K., Chhabra, S., Huang, W., Liu, H., Aref, A.R., et al. (2018). CDK4/6 Inhibition Augments Antitumor Immunity by Enhancing T-cell Activation. Cancer Discov. *8*, 216–233. https://doi.org/10.1158/2159-8290.CD-17-0915.
14. Lelliott, E.J., Mangiola, S., Ramsbottom, K.M., Zethoven, M., Lim, L., Lau, P.K.H., Oliver, A.J., Martelotto, L.G., Kirby, L., Martin, C., et al. (2021). Combined BRAF, MEK, and CDK4/6 Inhibition Depletes Intratumoral Immune-Potentiating Myeloid Populations in Melanoma. Cancer Immunol. Res. *9*, 136–146. https://doi.org/10.1158/2326-6066.CIR-20-0401.
15. van der Windt, G.J.W., Everts, B., Chang, C.H., Curtis, J.D., Freitas, T.C., Amiel, E., Pearce, E.J., and Pearce, E.L. (2012). Mitochondrial respiratory capacity is a critical regulator of CD8+ T cell memory development. Immunity *36*, 68–78. https://doi.org/10.1016/j.immuni.2011.12.007.
16. van der Windt, G.J.W., O'Sullivan, D., Everts, B., Huang, S.C.C., Buck, M.D., Curtis, J.D., Chang, C.H., Smith, A.M., Ai, T., Faubert, B., et al. (2013). CD8 memory T cells have a bioenergetic advantage that underlies their rapid recall ability. Proc. Natl. Acad. Sci. USA *110*, 14336–14341. https://doi.org/10.1073/pnas.1221740110.
17. Ferrick, D.A., Neilson, A., and Beeson, C. (2008). Advances in measuring cellular bioenergetics using extracellular flux. Drug Discov. Today *13*, 268–274. https://doi.org/10.1016/j.drudis.2007.12.008.
18. van der Windt, G.J.W., Chang, C.H., and Pearce, E.L. (2016). Measuring Bioenergetics in T Cells Using a Seahorse Extracellular Flux Analyzer. Curr. Protoc. Immunol. *113*, 3.16B.11–3.16B.14. https://doi.org/10.1002/0471142735.im0316bs113.
19. Gallego, G.M., Palmer, C., Orr, S., Bernier, L., Chen, P., Cho-Schultz, S., Deal, J.G., Dress, K., Edwards, M., Jalaie, M., et al. (2025). Discovery of Atirmociclib (PF-07220060): A Potent and Selective CDK4 Inhibitor. J. Med. Chem. *68*, 26085–26098. https://doi.org/10.1021/acs.jmedchem.5c02137.
20. Goel, S., Wang, Q., Watt, A.C., Tolaney, S.M., Dillon, D.A., Li, W., Ramm, S., Palmer, A.C., Yuzugullu, H., Varadan, V., et al. (2016). Overcoming Therapeutic Resistance in HER2-Positive Breast Cancers with CDK4/6 Inhibitors. Cancer Cell *29*, 255–269. https://doi.org/10.1016/j.ccell.2016.02.006.
21. Beavis, P.A., Henderson, M.A., Giuffrida, L., Mills, J.K., Sek, K., Cross, R.S., Davenport, A.J., John, L.B., Mardiana, S., Slaney, C.Y., et al. (2017). Targeting the adenosine 2A receptor enhances chimeric antigen receptor T cell efficacy. J. Clin. Invest. *127*, 929–941. https://doi.org/10.1172/JCI89455.

22. Chan, J.D., Scheffler, C.M., Munoz, I., Sek, K., Lee, J.N., Huang, Y.K., Yap, K.M., Saw, N.Y.L., Li, J., Chen, A.X.Y., et al. (2024). FOXO1 enhances CAR T cell stemness, metabolic fitness and efficacy. Nature *629*, 201–210. https://doi.org/10.1038/s41586-024-07242-1.

23. Giuffrida, L., Sek, K., Henderson, M.A., House, I.G., Lai, J., Chen, A.X.Y., Todd, K.L., Petley, E.V., Mardiana, S., Todorovski, I., et al. (2020). IL-15 Preconditioning Augments CAR T Cell Responses to Checkpoint Blockade for Improved Treatment of Solid Tumors. Mol. Ther. *28*, 2379–2393. https://doi.org/10.1016/j.ymthe.2020.07.018.

24. Lai, A.Y., Sorrentino, J.A., Dragnev, K.H., Weiss, J.M., Owonikoko, T.K., Rytlewski, J.A., Hood, J., Yang, Z., Malik, R.K., Strum, J.C., and Roberts, P.J. (2020). CDK4/6 inhibition enhances antitumor efficacy of chemotherapy and immune checkpoint inhibitor combinations in preclinical models and enhances T-cell activation in patients with SCLC receiving chemotherapy. J. Immunother. Cancer *8*, e000847. https://doi.org/10.1136/jitc-2020-000847.

25. Crompton, J.G., Sukumar, M., Roychoudhuri, R., Clever, D., Gros, A., Eil, R.L., Tran, E., Hanada, K.I., Yu, Z., Palmer, D.C., et al. (2015). Akt inhibition enhances expansion of potent tumor-specific lymphocytes with memory cell characteristics. Cancer Res. *75*, 296–305. https://doi.org/10.1158/0008-5472.CAN-14-2277.

26. Meyran, D., Terry, R.L., Zhu, J.J., Haber, M., Ziegler, D.S., Ekert, P.G., Trapani, J.A., Darcy, P.K., and Neeson, P.J. (2021). Early-phenotype CAR-T cells for the treatment of pediatric cancers. Ann. Oncol. *32*, 1366–1380. https://doi.org/10.1016/j.annonc.2021.07.018.

27. June, C.H., and Sadelain, M. (2018). Chimeric Antigen Receptor Therapy. N. Engl. J. Med. *379*, 64–73. https://doi.org/10.1056/NEJMra1706169.

28. Power, B.E., Caine, J.M., Burns, J.E., Shapira, D.R., Hattarki, M.K., Tahtis, K., Lee, F.T., Smyth, F.E., Scott, A.M., Kortt, A.A., and Hudson, P.J. (2001). Construction, expression and characterisation of a single-chain diabody derived from a humanised anti-Lewis Y cancer targeting antibody using a heat-inducible bacterial secretion vector. Cancer Immunol. Immunother. *50*, 241–250. https://doi.org/10.1007/s002620100192.

29. Scott, A.M., Geleick, D., Rubira, M., Clarke, K., Nice, E.C., Smyth, F.E., Stockert, E., Richards, E.C., Carr, F.J., Harris, W.J., et al. (2000). Construction, production, and characterization of humanized anti-Lewis Y monoclonal antibody 3S193 for targeted immunotherapy of solid tumors. Cancer Res. *60*, 3254–3261.

30. Westwood, J.A., Smyth, M.J., Teng, M.W.L., Moeller, M., Trapani, J.A., Scott, A.M., Smyth, F.E., Cartwright, G.A., Power, B.E., Hönemann, D., et al. (2005). Adoptive transfer of T cells modified with a humanized chimeric receptor gene inhibits growth of Lewis-Y-expressing tumors in mice. Proc. Natl. Acad. Sci. USA *102*, 19051–19056. https://doi.org/10.1073/pnas.0504312102.

31. John, L.B., Devaud, C., Duong, C.P.M., Yong, C.S., Beavis, P.A., Haynes, N.M., Chow, M.T., Smyth, M.J., Kershaw, M.H., and Darcy, P.K. (2013). Anti-PD-1 antibody therapy potently enhances the eradication of established tumors by gene-modified T cells. Clin. Cancer Res. *19*, 5636–5646. https://doi.org/10.1158/1078-0432.CCR-13-0458.

32. Haynes, N.M., Snook, M.B., Trapani, J.A., Cerruti, L., Jane, S.M., Smyth, M.J., and Darcy, P.K. (2001). Redirecting mouse CTL against colon carcinoma: superior signaling efficacy of single-chain variable domain chimeras containing TCR-zeta vs Fc epsilon RI-gamma. J. Immunol. *166*, 182–187. https://doi.org/10.4049/jimmunol.166.1.182.

33. Darcy, P.K., Haynes, N.M., Snook, M.B., Trapani, J.A., Cerruti, L., Jane, S.M., and Smyth, M.J. (2000). Redirected perforin-dependent lysis of colon carcinoma by ex vivo genetically engineered CTL. J. Immunol. *164*, 3705–3712. https://doi.org/10.4049/jimmunol.164.7.3705.

34. Michie, J., Beavis, P.A., Freeman, A.J., Vervoort, S.J., Ramsbottom, K.M., Narasimhan, V., Lelliott, E.J., Lalaoui, N., Ramsay, R.G., Johnstone, R.W., et al. (2019). Antagonism of IAPs Enhances CAR T-cell Efficacy. Cancer Immunol. Res. *7*, 183–192. https://doi.org/10.1158/2326-6066.CIR-18-0428.

35. Kearney, C.J., Lalaoui, N., Freeman, A.J., Ramsbottom, K.M., Silke, J., and Oliaro, J. (2017). PD-L1 and IAPs co-operate to protect tumors from cytotoxic lymphocyte-derived TNF. Cell Death Differ. *24*, 1705–1716. https://doi.org/10.1038/cdd.2017.94.

YMTHE, Volume *34*

Supplemental Information

CDK4/6 inhibition enhances

CAR-T cell therapy in solid tumors

Emily J. Lelliott, Jonathan Naddaf, Kun-Hui Lu, Katherine D. Cummins, Kelly M. Ramsbottom, David S. Reynolds, Michael Taylor, Krutika Ambani, Isabelle Munoz, Susan Jackson, Jessica Li, Cheok Weng Chan, Kara L. Britt, Paul A. Beavis, Shom Goel, and Jane Oliaro

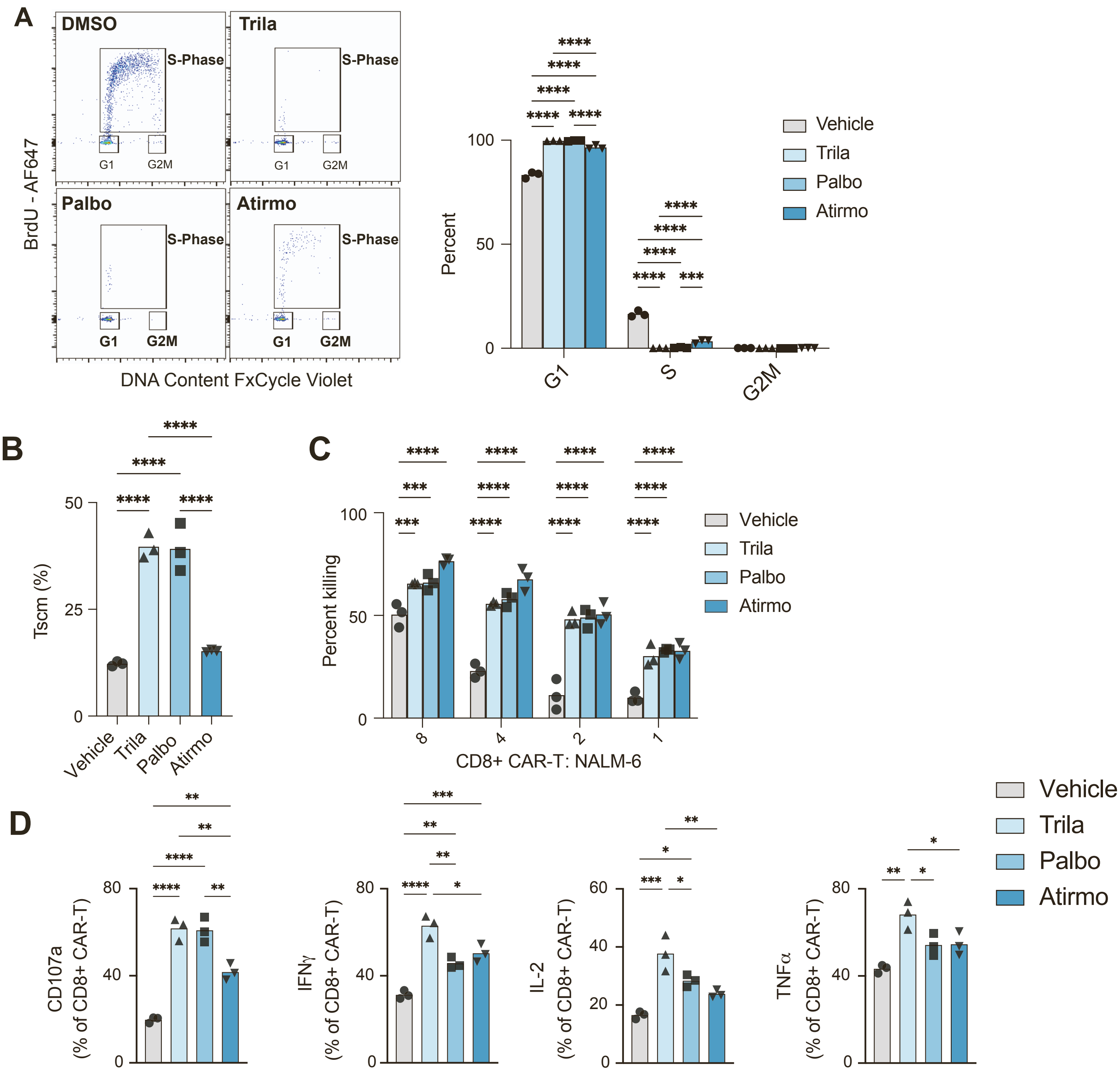

Figure S1. Human T cells were isolated from healthy donors, activated, and transduced to express a CD19-directed CAR. Transduced cells were then cultured for 6 days in trilaciclib, palbociclib, atirmociclib or DMSO (vehicle) as described in Figure 1A. (A) Cell cycle analysis using BrdU incorporation. BrdU was added to drug treated cells in culture 1 hour prior to harvest and analysis by flow cytometry. Comparisons were made using Ordinary two-way ANOVA Tukey's multiple comparisons test, n=3. (B) Drug-treated CAR-T cells were harvested and the frequency of stem cell memory T cells (Tscm) was determined by flow cytometry using gating shown in Figure 1A. Comparisons were made using Ordinary one-way ANOVA Tukey's multiple comparisons test, n=3. (C) CAR-T cell killing activity against NALM6 tumor cell targets. CAR-T cells were co-cultured with 51Cr-labelled NALM6 tumor cells at increasing ratios of CD8+ CAR-T cells to NALM6 tumor cells. Specific lysis was measured by percent chromium release and relative killing was calculated by normalising vehicle control to maximum killing of 100%. Comparisons made using Ordinary two-way ANOVA Tukey's multiple comparisons test, n=3. (D) Degranulation (CD107a) and cytokine production by CAR-T cells co-cultured with NALM6 tumor cells in vitro for 4 h. Fluor-conjugated CD107a-targeting antibody and Golgi plug was added to co-cultures before staining for analysis by flow cytometry. Comparisons made using Ordinary two-way ANOVA Tukey's multiple comparisons test, n=3. Error bars show +/- SEM. *$p<0.05$, **$p<0.01$, ***$p<0.001$, ****$p<0.0001$.

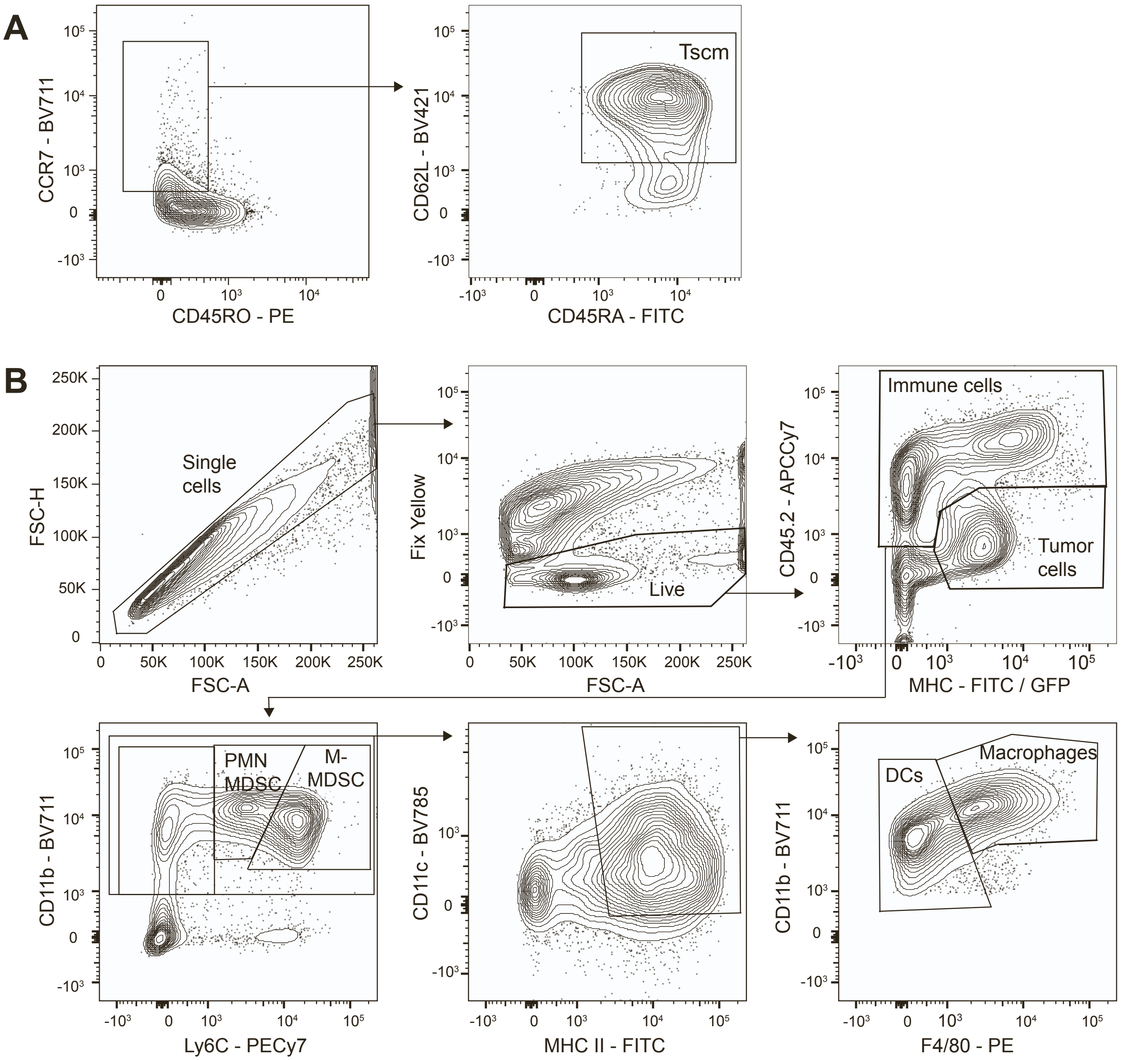

Figure S2. Flow cytometry gating. (A) Gating for human stem cell memory T cells (Tscm) first gated on CD8+ T cells. (B) Gating for tumor cells myeloid subsets from GFP+ E0771-hHER2 tumors.

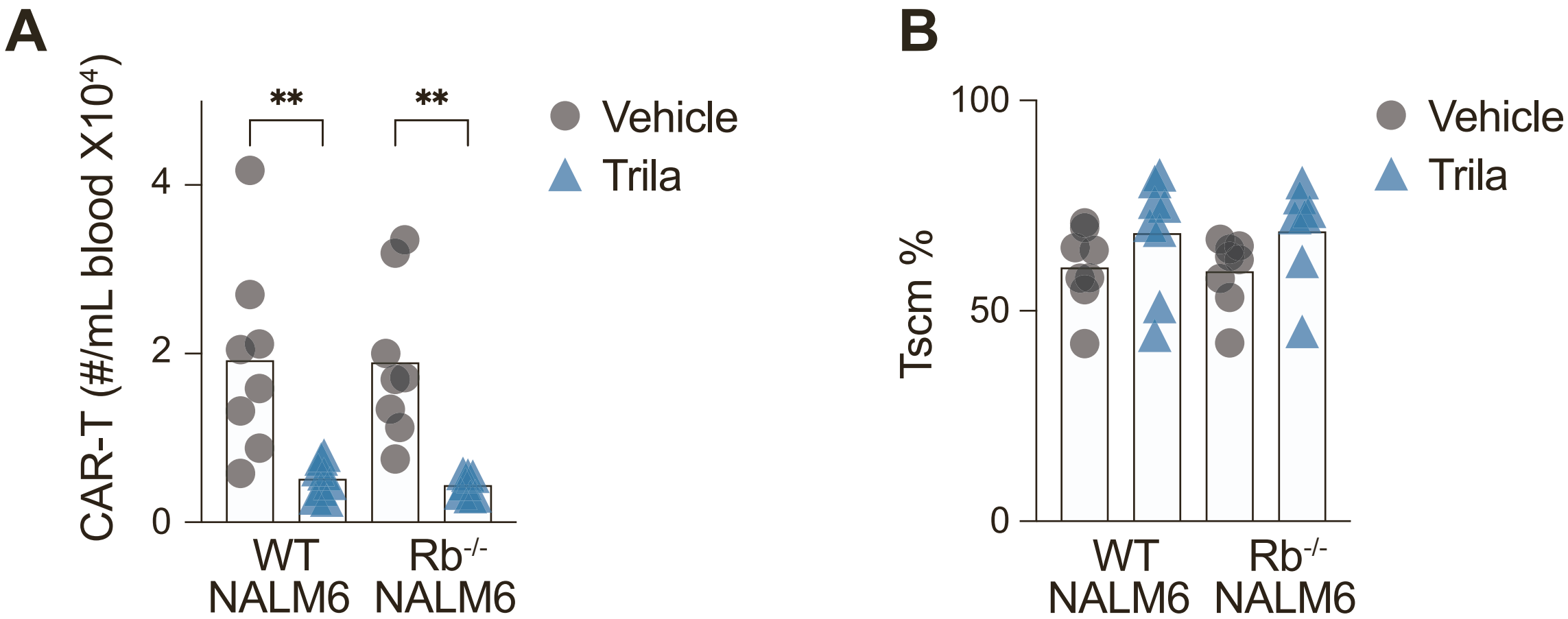

Figure S3. CD19-directed human CAR-T cells were transferred into NSG mice bearing wildtype (WT) or Rb deficient (Rb-/-) NALM6 blood cancer. Mice were treated with continuous 3 days on/4 days off trilaciclib regimen starting the day of CAR-T cell transfer. Blood was collected after two cycles of treatment. (A) CAR-T cell numbers and (B) frequency of CD8+ stem cell memory CAR-T cells (Tscm) were determined by flow cytometry. Comparisons made using Ordinary one-way ANOVA Tukey's multiple comparisons test, n=3. Error bars show +/- SEM. **p<0.01.

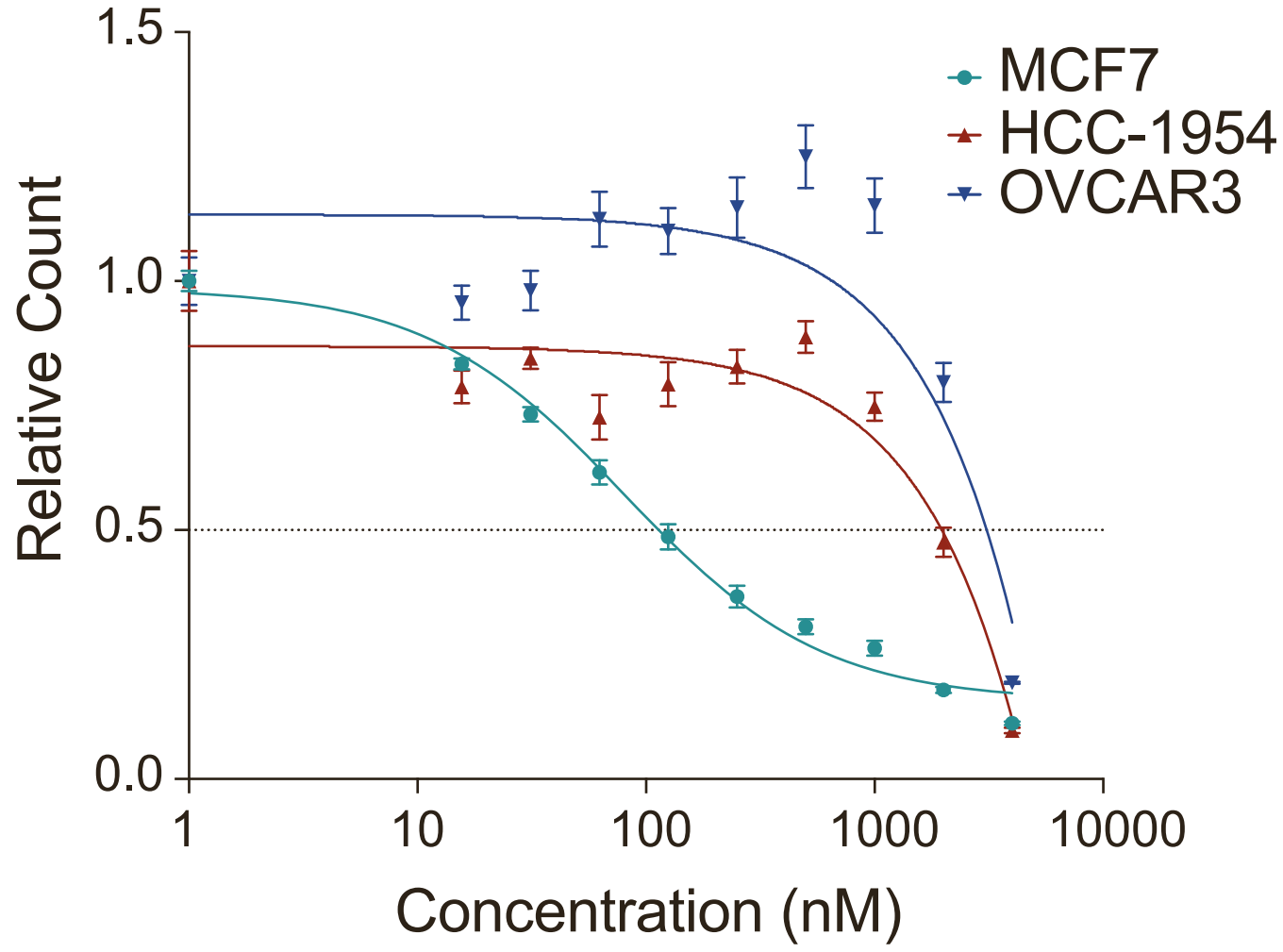

Figure S4. MCF7, OVCAR3, and HCC1954 cells were treated with a ten-point dose concentration from 15.625 nmol/L to 4000 nmol/L trilaciclib for 72 hours to determine dose response.

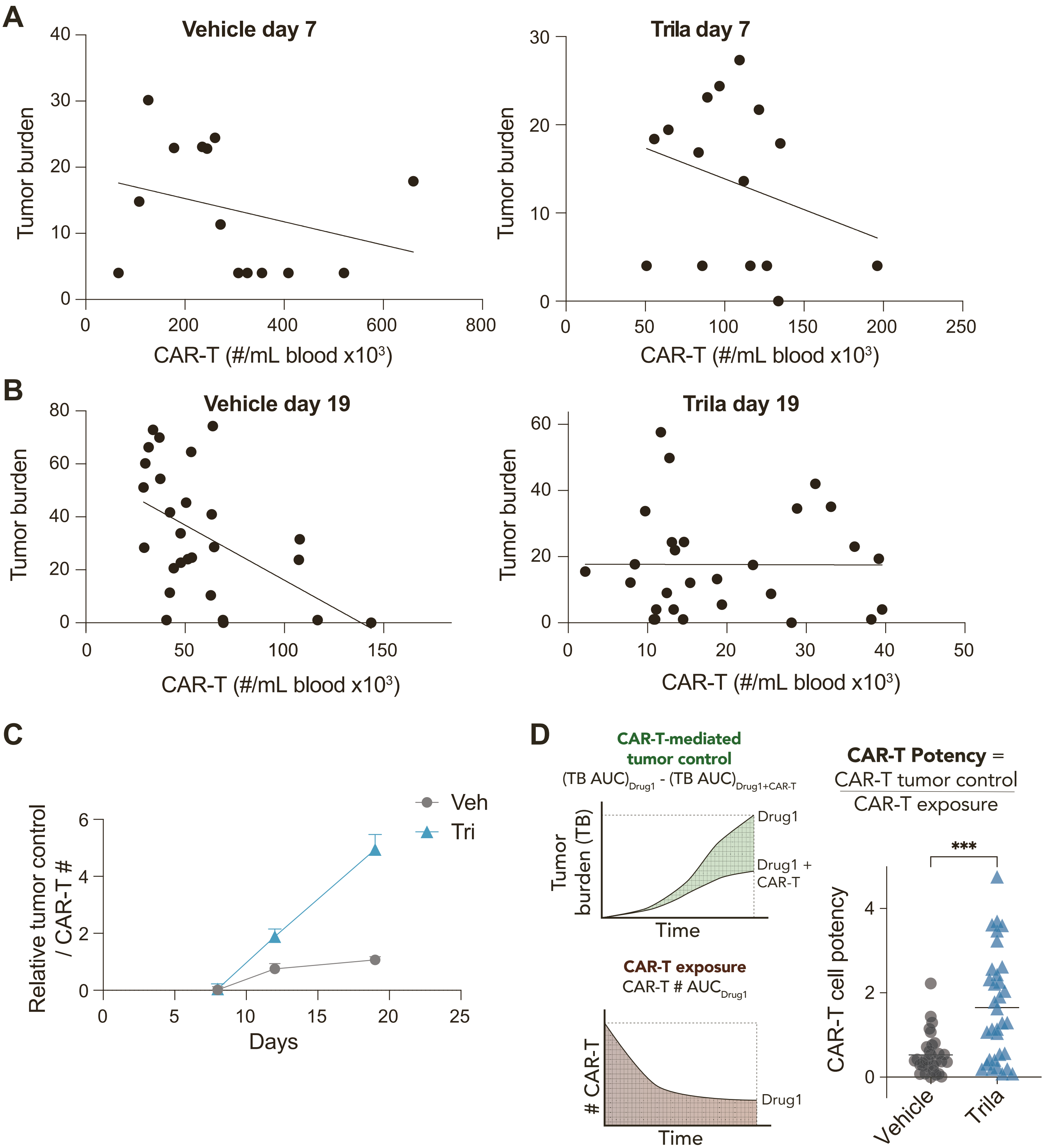

Figure S5. (A-B) Correlation between circulating CAR-T cell numbers and tumor burden at indicated time points in vehicle- and trilaciclib-treated E0771-hHER tumor bearing mice receiving hHER2-directed CAR-T cells. (C) Per-cell CAR-T anti-tumor potency over time, calculated by normalizing tumor burden in each combination-treated mice to the average tumor burden of matched drug-only controls, and dividing by CAR-T cell numbers in the blood for the same mouse at the corresponding time point. (D) Integrated measure of overall CAR-T cell potency, calculated as cumulative CAR-T–mediated tumor control (difference in tumor AUC relative to drug-only controls) divided by cumulative CAR-T cell exposure (CAR-T cell AUC). Comparisons made using Mann-Whitney nonparametric test, n=31-33, ***$p<0.001$.

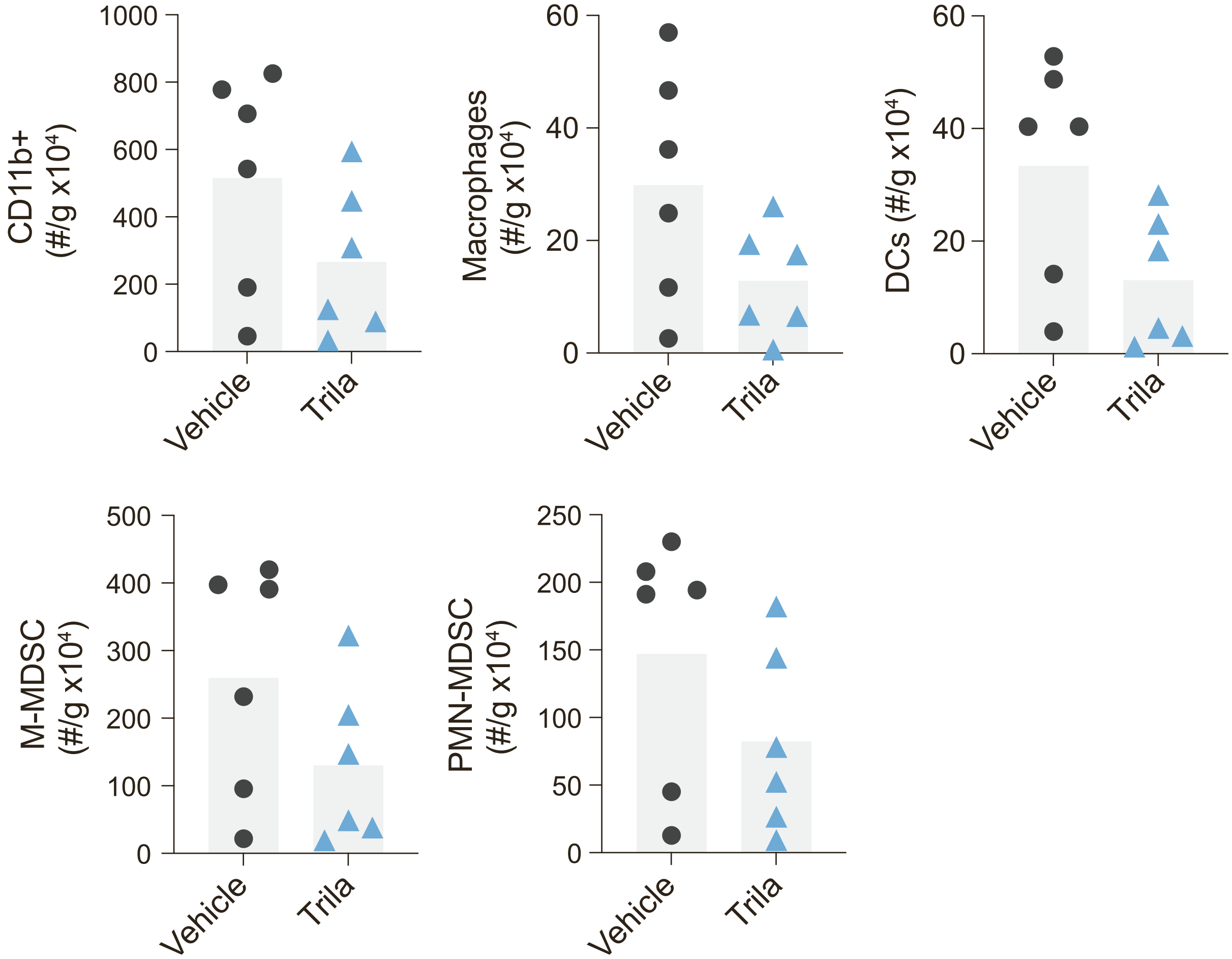

Figure S6. HER2-directed mouse CAR-T cells were transferred into immunocompetent C57BL/5 mice 5 days post mammary fat pad inoculation with the mouse breast cancer cell line E0771-hHER2. Mice were treated with continuous 3 days on/4 days off trilaciclib regimen starting the day of CAR-T cell transfer. Tumors were harvested following two cycles of treatment. Total number of myeloid populations was determined by flow cytometry using gating shown in Figure S1B. No statistically significant differences observed by Mann-Whitney nonparametric t-test.
